# Cognitive impairment within and beyond the FTD spectrum in ALS: development of a complementary cognitive screen

**DOI:** 10.1007/s00415-025-13006-2

**Published:** 2025-03-13

**Authors:** Annebelle Michielsen, Kevin van Veenhuijzen, Fenna Hiemstra, Ilse M. Jansen, Boaz Kalkhoven, Jan H. Veldink, Esther T. Kruitwagen, Michael van Es, Martine J. E. van Zandvoort, Leonard H. van den Berg, Henk-Jan Westeneng

**Affiliations:** 1 Department of Neurology, UMC Utrecht Brain Center, University Medical Center Utrecht, G03.232, P.O. Box 85500, 3508 GA Utrecht, The Netherlands; 2 Department of Experimental Psychology, Utrecht University, Utrecht, The Netherlands; 3 Department of Rehabilitation, Physical Therapy Science & Sports, UMC Utrecht Brain Center, University Medical Center Utrecht, Utrecht, The Netherlands; 4 Center of Excellence for Rehabilitation Medicine, UMC Utrecht Brain Center, University Medical Center Utrecht, and de Hoogstraat Rehabilitation, Utrecht, The Netherlands

**Keywords:** Amyotrophic lateral sclerosis, Motor neuron disease, Cognition, Frontotemporal dementia, Cognitive screening instrument, Social cognition

## Abstract

**Objective:**

To investigate cognitive impairments in amyotrophic lateral sclerosis (ALS), extending both within and beyond the established frontotemporal dementia (FTD) spectrum, using the Complementary Cognitive ALS Screen (C-CAS).

**Methods:**

The C-CAS, designed to complement the Edinburgh cognitive and behavioural ALS screen (ECAS), explores cognitive (sub)domains not investigated by the ECAS. Normative data were collected, and models adjusted for age, sex, and education level were developed. Item scores below the 5th percentile in controls were considered abnormal. A sum score was constructed, and C-CAS impairments were compared between ALS patients and controls, and to ECAS impairments.

**Results:**

Data from 314 controls and 184 ALS patients were analyzed. The C-CAS is feasible, well-tolerated, and takes 15–20 min to complete. ALS patients performed worse across all 12 items. Within the FTD spectrum, impairments in social cognition, inhibition, and cognitive flexibility were identified in up to 16%, 14%, and 12% of ALS patients, respectively, with minimal overlap with ECAS impairments. Beyond the FTD spectrum, impairments were found in visuoconstruction, incidental non-verbal memory and body orientation (13% each), with minimal overlap with ECAS memory or visuospatial impairments. Overall, 24% of the ALS patients obtained an abnormal C-CAS sum score. Compared to the ECAS, the C-CAS detected additional impairments in 15% of ALS patients. Item-specific and sum score results based on normative data can be accessed at (https://apps4mnd.com/ccas/).

**Interpretation:**

We identified cognitive impairments in ALS within and beyond the FTD spectrum not captured by existing screening tools. The C-CAS complements the ECAS, improving personalized counseling and research stratification in ALS.

**Supplementary Information:**

The online version contains supplementary material available at 10.1007/s00415-025-13006-2.

## Introduction

Amyotrophic lateral sclerosis (ALS) is a progressive neurodegenerative disease characterized by muscle weakness, spasticity, and a range of non-motor symptoms, including cognitive and behavioral changes [1]. Up to 50% of ALS patients exhibit impairments in cognition and behavior that frequently mirror those observed in frontotemporal dementia (FTD) [2–4]. Consequently, classifications such as ALS with cognitive impairment (ALS-ci), ALS with behavioral impairment (ALS-bi), or a combination (ALS-cbi) have been established [2]. Up to 15% of cases present with both ALS and FTD (ALS-FTD) [2, 3, 5]. Recognizing and characterizing cognitive and behavioral function in ALS is crucial, not only for personalized counseling and guiding treatment decisions, but also for developing targeted therapies [3, 6]. Moreover, it may enhance our understanding of the disease’s pathophysiology.

Screening instruments play an important role in identifying cognitive and behavioral changes. The Edinburgh Cognitive and Behavioural ALS Screen (ECAS) is a valuable tool designed to identify these specific changes in ALS and distinguish them from other disorders [7]. It screens for deficits in five cognitive domains (executive function, language, verbal fluency, memory and visuospatial function), and assesses behavioral abnormalities through caregiver interviews. A recent neuroimaging study has uncovered distinct ALS subtypes with unique neurodegenerative patterns [8]. Besides the well-known pure motor and frontotemporal subtype, a new phenotype has emerged, characterized by involvement of posterior cingulate, parietal, temporal, and cerebellar regions (CPT subtype). The frontotemporal and CPT subtype exhibited similar cognitive deficits on cognitive screening, despite distinct patterns of neurodegeneration underlying each subtype. Due to its focus on cognitive changes within the FTD spectrum, the ECAS may be less sensitive to deficits associated with posterior parietal brain functions [8]. Identifying these potential impairments beyond the FTD spectrum is crucial, as they may require different guidance compared to frontotemporal problems.

Furthermore, conceptual advances have led to a better understanding of social cognition as a separate domain from executive function and an increased recognition of the complexity within these domains. The ECAS combines executive function and social cognition into one domain, but dissociating these cognitive functions may offer a more fine-grained understanding of the specific dysfunctions in the context of ALS. Moreover, both social cognition and executive function comprise several subdomains, including emotion recognition, theory of mind, inhibition, and cognitive flexibility [2, 9]. Exploring these areas may uncover impairments essential for effective communication, education, and support for patients and their caregivers.

Therefore, this study uses a complementary cognitive screen to investigate the presence of cognitive deficits in ALS patients that may not be captured by existing screening tools, extending both within and beyond the established FTD spectrum. Through a cross-sectional, prospective case–control study, we developed the Complementary Cognitive ALS Screen (C-CAS), defined normative values in control data, studied impairments in ALS patients, and compared the screen’s performance with the ECAS.

## Methods

### Study design and participants

We conducted a cross-sectional prospective case–control study to investigate cognitive functions, potentially going beyond the current scope of existing screening tools in ALS. As part of this investigation, we developed a brief neuropsychological screening instrument, intended to complement the ECAS. Participants were recruited between May 2022 and January 2024 from an ongoing population-based study in the Netherlands [10]. Inclusion criteria were Dutch as a first language, and absence of pre-existing conditions that could influence test performance: learning and/or reading disabilities, dyslexia, history of stroke, epilepsy, psychosis or schizophrenia, use of antipsychotics, or substance abuse. In addition, controls were required to have neither writing disabilities nor any neurodegenerative disease.

### Demographic and clinical assessment

Demographic information was collected for all participants. Clinical characteristics of patients were collected at time of diagnosis and included information about site of disease onset, presence of bulbar symptoms, diagnosis of frontotemporal dementia (FTD), and survival status. Survival was defined as time between symptom onset and non-invasive ventilation for more than 23 h per day, tracheostomy, or all-cause mortality. Symptom duration was set as time between symptom onset and date of cognitive assessment. Daily functioning and disease progression rate (∆FRS) were determined using the revised ALS functional rating scale (ALS-FRS-R) [11]. ∆FRS was calculated using the formula (48 minus total ALSFRS-R score) / symptom duration (in months). The education level, according to the International Standard Classification of Education (ISCED 2011), was registered for all participants.

### Development of C-CAS

The C-CAS comprises items targeted at a wide spectrum of cognitive functions in ALS. Most items are based on established cognitive paradigms and tailored to accommodate the motor limitations of ALS patients and shorten the screening time. The final battery comprised 12 items tapping into 6 cognitive domains. These domains include functions commonly affected in the spectrum of the behavioral variant of FTD, such as social cognition and subdomains of executive function, like inhibition and cognitive flexibility. In addition, to assess changes in functions associated with posterior parietal brain regions, the battery included domains not typically associated with the FTD spectrum, such as visuoconstruction, incidental non-verbal memory and body orientation.

Here, we outline the 12 C-CAS items per domain. To assess social cognition, we incorporated three items that tap into different aspects of this domain: emotion recognition from facial expressions and two items that address theory of mind (simple and complex). These items were developed by building upon experience with emotion recognition using schematic faces and the hinting task [12, 13]. To evaluate inhibition, defined as the ability to suppress pre-potent behavior that is no longer required or has become inappropriate, we included items tapping into interference control, conflicting instructions, and action restraint. Interference control refers to the mental ability to focus on a specific task or stimulus and exclude distractions, a crucial cognitive function that allows us to concentrate, resist impulses, and filter out background noise. In the C-CAS, we assess interference control using response time, represented as a ratio, and error rate on a paradigm adapted from the color–word interference test (also known as the ‘Stroop task’). Conflicting instructions and action restraint are assessed using items derived from the conflicting instructions and go/no-go tasks as found in the Frontal Assessment Battery. [14, 15] To focus purely on inhibition while limiting the impact of reactive control problems, normal/abnormal scores were determined for the action restraint item if participants successfully completed the conflicting instructions item [16]. Cognitive flexibility includes two items assessing the ability to switch between mental sets and adapt to changing demands. They are adapted from the color–word interference task and Bergen right–left discrimination task [14, 17]. Furthermore, items targeting domains usually spared within the FTD spectrum were added to specifically investigate the involvement of posterior parietal brain regions. Visuoconstruction includes the copy score from the Rey–Osterrieth complex figure test, which measures the ability to visually perceive, analyze, and reproduce a complex geometric design [18]. Incidental non-verbal memory is measured by the recall score, investigating the ability to retain and reproduce the same design from memory after a delay, without the instruction to memorize (incidental). Finally, body orientation comprises an item adapted from the Bergen right–left discrimination task, combining front and back view into a single score to assess participants’ ability to identify body parts, regardless of their relative position [17]. The C-CAS form, manual and appendices are available as supplementary material.

Designed to adapt to motor symptoms, the C-CAS allows most patients to complete all items regardless of bulbar or hand motor disabilities. Only those with severe bulbar dysfunction resulting in incomprehensible speech may be unable to perform the interference control items (3/12 items). Similarly, patients with severe hand motor impairments may not be able to complete the Rey copy and recall items (2/12 items). In both cases, the remaining items of the C-CAS can still be completed, a sum score can be calculated, and cut-offs accounting for the missing data can be applied. The battery was administered to both ALS patients and controls either by neuropsychologists or by experienced clinicians under the supervision of a neuropsychologist. Administration of the C-CAS by a diverse team of clinicians ensures its broad applicability across various healthcare settings.

### Cognitive and behavioral assessment using existing screening instruments

Routine cognitive and behavioral assessments, including the ECAS, ALS-FTD Questionnaire (ALS-FTD-Q) and Hospital Anxiety and Depression Scale (HADS), were administered within 3 months of the C-CAS [19, 20]. The five cognitive domain scores of the ECAS (language, fluency, executive, memory, and visuospatial) were characterized as normal or abnormal using age- and education-corrected normative data from the Dutch version of the ECAS [21]. The presence of behavioral change was assessed through the caregiver interview of the ECAS and the ALS-FTD-Q. Occurrence of cognitive impairment (ALS-ci), behavioral impairment (ALS-bi) or both (ALS-cbi) were determined in ALS patients according to the revised Strong criteria [2].

### Statistical analyses

All analyses were executed using R (version 4.2.2). Clinical and demographic variables were compared using a Mann–Whitney U-test for continuous variables and Fisher exact test for categorical variables.

#### Derivation of normative data

To establish normative data for controls, we employed a multi-step approach to identify the most suitable predictive model for each item. We evaluated the following models and regression equations, including shifted lognormal, negative binomial, Poisson, and ordinal regression models. Age, sex, and education were considered as co-variables to adjust these models. Using backward elimination, we retained co-variables that significantly improved a model, as indicated by a greater than three-point reduction in Akaike Information Criteria (AIC). For the negative binomial and Poisson regression, outcome variables were inverted (i.e., higher scores representing worse performance). Supplementary Table 1 provides an overview of the characteristics of each regression model for C-CAS items, including the selected co-variables. Percentile ranks of residuals were calculated per cognitive item. The residual corresponding to the lowest 5th percentile rank in controls was selected as the cut-off score. The normative cut-off values for residual scores per item are presented in Supplementary Table 2.

#### Impairments in ALS based on C-CAS

We applied the models developed with control data to ALS patients and compared their observed scores with the expected scores based on the normative control data. This analysis generated residual values for each patient, indicating deviations from typical performance as shown by controls. Items with residuals below the specified cut-off, as delineated in Supplementary Table 2, were categorized as impaired. Subsequently, the frequency of impairments per item was compared between ALS patients and controls. In addition, a comparison was made between ALS patients and controls regarding the number of impaired items and impaired cognitive domains within the entire C-CAS. A domain was classified as impaired if at least one impaired item was present within that domain.

#### Exploring overlap and interrelationships of C-CAS items

Venn diagrams were created to analyze the overlap in abnormal scores within each C-CAS domain. This visualized the unique contribution of each item by identifying impairments missed by other items and capturing shared deficits. Furthermore, pairwise correlations between individual items of the C-CAS were calculated separately for ALS patients and controls to explore the interrelatedness of items.

#### C-CAS sum score

Using the residuals of the 12 individual items, a separate model was built to create a single score (sum score), which is a representation of overall cognitive performance within the C-CAS. After normalizing the residuals per item (to zero mean and standard deviation one), a multivariate normal distribution was fitted to the residuals of the 12 items. Each individual’s distance from the center of the multivariate normal distribution was multiplied by its multivariate inverse-variance weighted mean to encode both deviance (from the center) and direction (towards residual scores reflecting impairment) in the sum score. This score can be calculated if one item of the C-CAS is absent or if specific combinations, common in ALS patients with motor involvement, are missing. For patients with severe bulbar symptoms, this includes items 4, 5, and 8. For patients with severe motor symptoms in the hands, this includes items 10 and 11. The sum scores of controls were percentile-ranked, and cut-off values were determined for the lowest 1st, 2nd, 5th, 10th, 25th, 50th, 75th percentiles for a more continuous performance measure on the sum score. These cut-off values were then applied to the sum scores of ALS patients, and the frequency of subjects scoring below these cut-offs was calculated.

#### Comparison of impairments on ECAS and C-CAS

To assess whether the C-CAS provides complementary information beyond what the ECAS already captures, comparisons of impairments between ECAS and C-CAS were performed. Impairments on the C-CAS sum score were compared to impairments on the ECAS total, ECAS specific, and ECAS nonspecific scores. In addition, given that some of the new items expanded upon domains assessed by the ECAS (executive function and social cognition), impairments on these items were compared. As the ECAS classifies its social cognition item (eye-gaze task) within the broader executive function domain score, direct comparisons between social cognition items were not possible. To overcome this, we established normative data and a cut-off score for the ECAS eye-gaze task, mirroring our approach with the C-CAS items, using control participant data. Finally, impairments on domains affected beyond the FTD spectrum (visuoconstruction and incidental non-verbal memory) were compared to corresponding ECAS nonspecific domains (visuospatial function and memory). Since body orientation could not be directly linked to a specific ECAS domain, a direct comparison for this domain was not possible.

#### Sensitivity analysis

To account for the potential influence of anxiety and depression on test performance, we evaluated their impact on the sum score in ALS patients. In a first linear model, we assessed the relationship between the C-CAS sum score and the HADS anxiety subscale, and in a second linear model, the relationship between this sum score and the HADS depression subscale. We considered results statistically significant if *p* < 0.05.

## Results

### Demographic and clinical characteristics

In total, 314 controls and 184 ALS patients participated in the study. The demographic and clinical characteristics of the ALS patients and controls are summarized in Table 1. The cohort of ALS patients in this study is representative of the general ALS population in the Netherlands, with cognitive and behavioral impairments similar to previous studies [21–23]. There were no significant differences in demographic characteristics, including education level, between ALS patients and controls.

**Table 1:** Demographic and clinical characteristics

	ALS	Control	Missing data, %
N	184	314	
Female	54 (29)	99 (32)	0
Age at C-CAS, years	65 (60–73)	67 (61–73)	0
Level of education ISCED	3 (3–6)	4 (3–6)	0
Bulbar onset	45 (25)		1
ALS-FRS-R score	40 (36–43)		5
∆FRS^a^, points/month	0.59 (0.31–0.98)		5
Symptom duration^b^, months	15 (9–26)		1
*C9orf72* repeat expansion	19 (13)		18
ALS-ci / ALS-bi / ALS-cbi / FTD	18 (12) / 15 (10) / 8 (6) / 5 (3)		15 / 20 / 21 / 19
ECAS cognitive impairment			
Total score	22 (14)		15
Specific score	20 (13)		15
Executive domain	13 (8)		15
Language domain	5 (3)		15
Fluency domain	16 (10)		15
Nonspecific score	10 (6)		14
Visuospatial domain	7 (4)		14
Memory domain	9 (6)		14
ALS-FTD-Q total score	4 (1–13)		20
Mild to severe behavioral change (> 22)	10 (7)		20
Severe behavioral changes (> 29)	9 (6)		20
HADS possible/suspected anxiety disorder	24 (18) / 14 (11)		29
HADS possible/suspected depression	16 (12) / 6 (5)		29

Overview of demographic and clinical characteristics. Data are count (%) or median (IQR)

^a^∆FRS was calculated as (48 minus total ALSFRS-R score)/(symptom duration in months)

^b^Symptom duration was calculated as time between symptom onset and date of C-CAS

Patients exhibiting cognitive and/or behavioral impairment and meeting the specified criteria, can be classified into one of the ALS-ci, ALS-bi, ALS-cbi or FTD categories. *ALS* amyotrophic lateral sclerosis *ISCED* International Standard Classification of Education (2011) *ALS* amyotrophic lateral sclerosis *ISCED* International Standard Classification of Education (2011) *ALS-FRS-R* revised ALS functional rating scale *FTD* frontotemporal dementia *ALS-ci* ALS with cognitive impairment *ALS-bi* ALS with behavioral impairment *ALS-cbi* ALS with cognitive and behavioral impairment *ECAS* Edinburgh Cognitive and Behavioural ALS Screen *ALS-FTD-Q* ALS-FTD Questionnaire *HADS* Hospital Anxiety and Depression Scale

### Feasibility of C-CAS

Patients tolerated the novel screening battery well, with less than 7% experiencing motor symptoms that prevented them from completing items requiring bulbar (3/12 items) or hand (2/12 items) motor function. The average time to complete the C-CAS was 15–20 min.

### C-CAS performance

On all items of the C-CAS, 4–5% of the controls scored below cut-off, reflecting the chosen cut-off at the 5th percentile (Table 2). Across all six domains, a higher percentage of ALS patients performed below cut-off compared to controls. The proportion of patients scoring below cut-off on each item ranged from 8% (Item 1: emotion recognition) to 16% (Item 3: theory of Mind complex). The sum score from the C-CAS was impaired in 24% of patients, contrasting with 5% of controls (in accordance with the chosen cut-off at the 5th percentile). Moreover, as demonstrated in Table 3, the C-CAS sum score showed that ALS patients had lower scores than controls, with the most notable difference observed in the lowest percentile rank (15% vs. 1%). Impairments in three or more items were rare among controls (2%), but more prevalent in ALS patients (17%), as shown in Table 4. Similarly, impairments in three or more domains were more commonly observed in ALS patients (14%) than in controls (2%).

**Table 2:** C-CAS performance in ALS patients and controls

Domain	ALS patients		Controls	
Social cognition				
Item 1: Emotion recognition	15/184	8%	16/313	5%
Item 2: Theory of Mind simple	26/184	14%	16/313	5%
Item 3: Theory of Mind complex	26/166	16%	14/277	5%
Executive function: Inhibition				
Item 4: Interference control time	17/172	10%	16/311	5%
Item 5: Interference control errors	20/174	11%	16/313	5%
Item 6: Conflicting instructions	15/175	9%	12/304	4%
Item 7: Action restraint	20/144	14%	14/272	5%
Executive function: Cognitive flexibility				
Item 8: Cognitive flexibility interference control	20/173	12%	16/313	5%
Item 9: Cognitive flexibility body-related	17/184	9%	17/310	5%
Visuoconstruction				
Item 10: Rey copy	23/175	13%	16/314	5%
Incidental non-verbal memory				
Item 11: Rey recall	22/173	13%	16/310	5%
Body orientation				
Item 12: Body orientation total	24/184	13%	16/312	5%
Sum score	**44/184**	**24%**	**15/309**	**5%**

Frequency and percentage of ALS patients and controls with cognitive deficits according to C-CAS cut-off scores. Data are presented as counts/number of subjects assessed (%). Domains are displayed in a hierarchical order, with those within the FTD spectrum appearing before those beyond the FTD spectrum. Item 7 has fewer participants with available data, because we only analyzed Item 7 after achieving maximal performance on Item 6

*ALS* amyotrophic lateral sclerosis *C-CAS* Complementary Cognitive ALS Screen

**Table 3:** C-CAS sum score percentiles

Percentile	Sum score	ALS patients	Controls
< 1e	< − 13.87	27 (15%)	4 (1%)
< 2e	< − 12.96	31 (17%)	6 (2%)
< 5e	< − 8.34	44 (24%)	15 (5%)
< 10e	< − 5.45	58 (32%)	31 (10%)
< 25e	< − 2.59	81 (44%)	76 (25%)
< 50e	< − 0.19	107 (58%)	154 (50%)
< 75e	< 2.55	147 (80%)	231 (75%)

The frequency of ALS patients and controls scoring below different percentiles of the sum score of the C-CAS. Data are expressed as counts (%). *ALS* amyotrophic lateral sclerosis *C-CAS* Complementary Cognitive ALS Screen

**Table 4:** Number of impaired items and domains in ALS and controls

	ALS patients	Controls
Number of impaired items		
≥ 9	1 (1%)	–
≥ 8	1 (1%)	–
≥ 7	4 (2%)	–
≥ 6	5 (3%)	–
≥ 5	7 (4%)	2 (1%)
≥ 4	14 (8%)	4 (1%)
≥ 3	32 (17%)	7 (2%)
≥ 2	67 (36%)	43 (14%)
≥ 1	113 (61%)	129 (41%)
Number of impaired domains		
≥ 6	1 (1%)	–
≥ 5	6 (3%)	–
≥ 4	12 (7%)	3 (1%)
≥ 3	25 (14%)	7 (2%)
≥ 2	62 (34%)	35 (11%)
≥ 1	113 (61%)	129 (41%)

Frequency and percentage of impaired C-CAS items and domains in ALS patients and controls. A domain was classified as impaired if at least one impaired item was present within that domain. Data are expressed as counts (%). *ALS* amyotrophic lateral sclerosis *C-CAS* Complementary Cognitive ALS Screen

### C-CAS item overlap and interrelationships

**Fig. 1:**
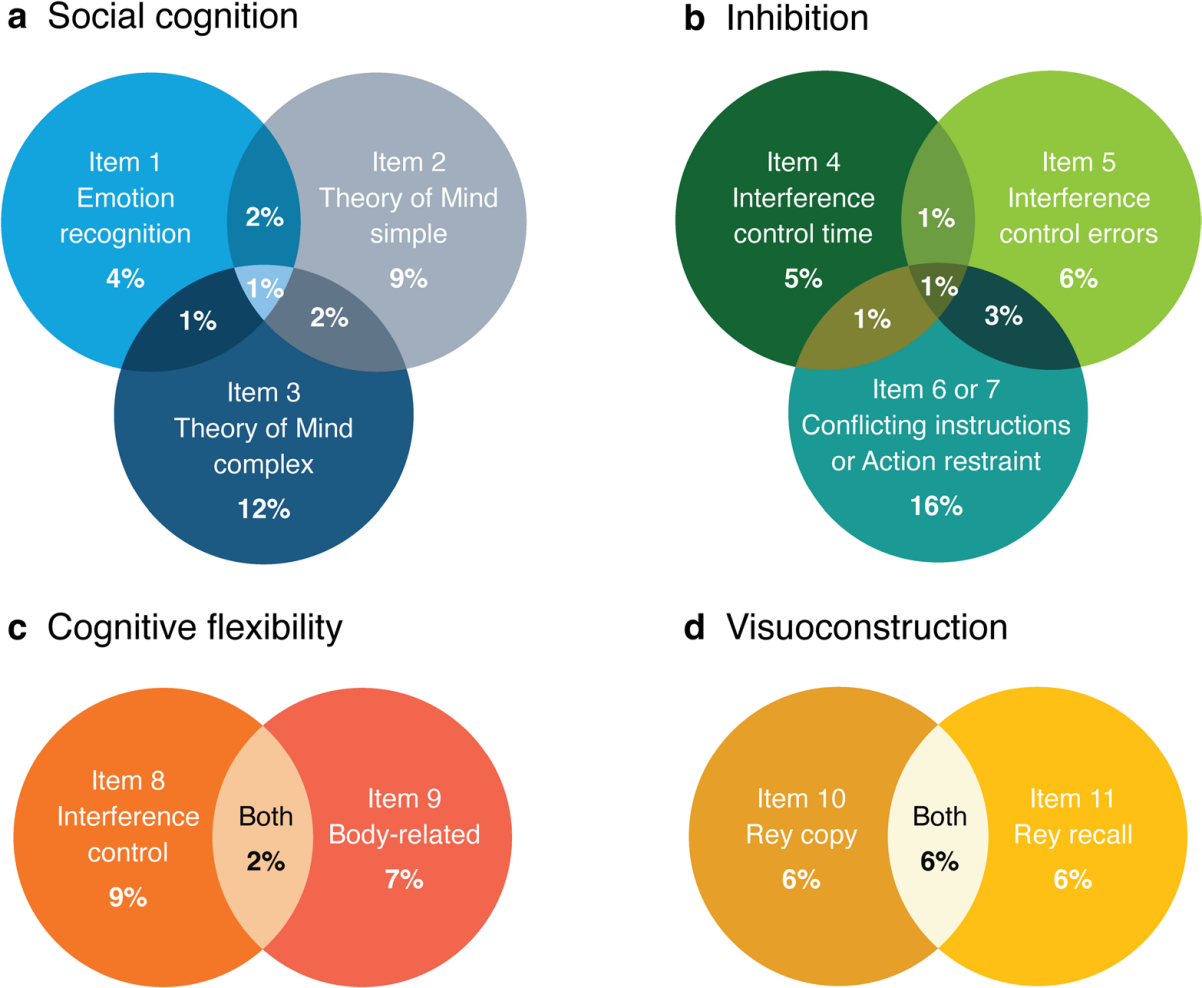
Overlap of impairments per item within each domain of the C-CAS Venn diagrams illustrating overlap of impairments per item within each domain **(a – d)** of the C-CAS. The percentages represent the proportion of patients with impaired scores. Items 6 and 7 are combined in diagram **(b)**, because Item 7 is present only when Item 6 performance is maximal. The percentages in the “Item 6 or 7” circle represent the proportion of patients with impaired scores on Item 6 or 7. In diagram **(d),** impairments in Item 10 and 11 are compared, as both involve the Rey figure (although Item 11 also involves memory). Since the domain body orientation comprises only one item (12), no comparisons are made within this domain. Due to missing data, there may be slight variations in percentages compared to those presented in Tables 1 and 2. *ECAS* Edinburgh Cognitive and Behavioural ALS Screen *C-CAS* Complementary Cognitive ALS Screen

The overlap between impairments of individual items within each domain of the C-CAS ranged from 1 to 6% (Fig. 1). In addition, the correlation coefficient between individual items of the C-CAS, excluding the correlations involving the sum score, was consistently below 0.20, both within and across domains (Supplementary Fig. 1), indicating the unique contribution of each item.

### Comparison of impairments in ECAS and C-CAS

**Fig. 2:**
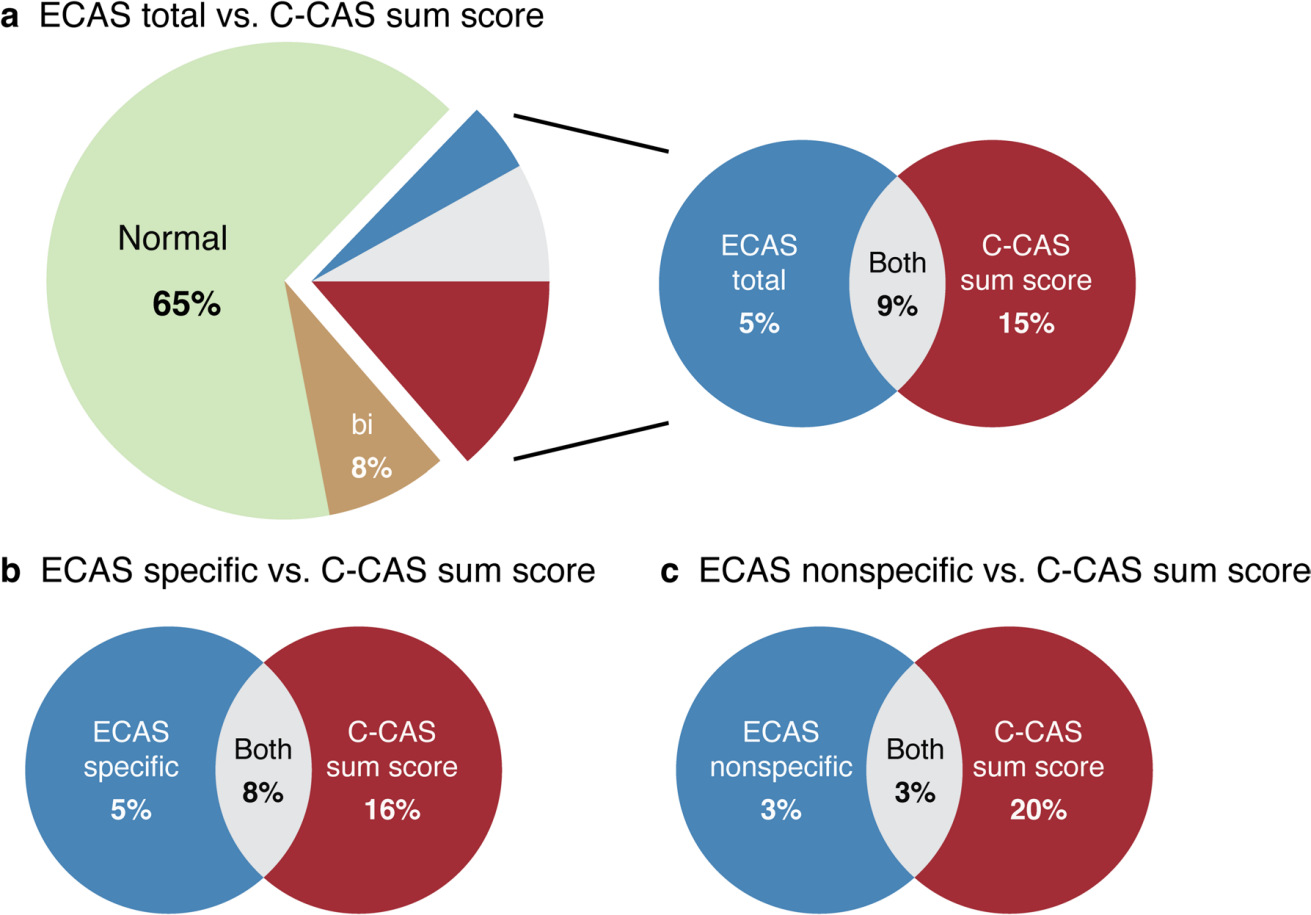
Comparison of impairments in ECAS and C-CAS sum scores The proportion of ALS patients with normal and impaired scores on ECAS and C-CAS. The Venn diagrams show the overlap of impaired ECAS total score **(a)**, ECAS specific score **(b)** and ECAS nonspecific score **(c)** compared to impaired C-CAS sum score. In **(a), ‘**bi’ refers to the proportion of patients with behavioral impairments with a normal score on both the ECAS total score and C-CAS sum score. Due to missing data, there may be slight variations in percentages compared to those in Tables 1 and 2. *ECAS* Edinburgh Cognitive and Behavioural ALS Screen *C-CAS* Complementary Cognitive ALS Screen

The C-CAS sum score was impaired in 15% of ALS patients with a normal ECAS total score, whereas the ECAS identified cognitive impairments in 5% of ALS patients with a normal C-CAS sum score (Fig. 2a). 9% of ALS patients exhibited abnormalities in both C-CAS sum score and ECAS total score. 73% of ALS patients obtained normal scores on both screening instruments (65% when also considering the absence of behavioral changes). The C-CAS sum score identified cognitive impairments that had not been noticed by the ECAS specific score in 16% of ALS patients (Fig. 2b), and impairments that were not detected by the ECAS nonspecific score in 20% of patients (Fig. 2c).

**Fig. 3:**
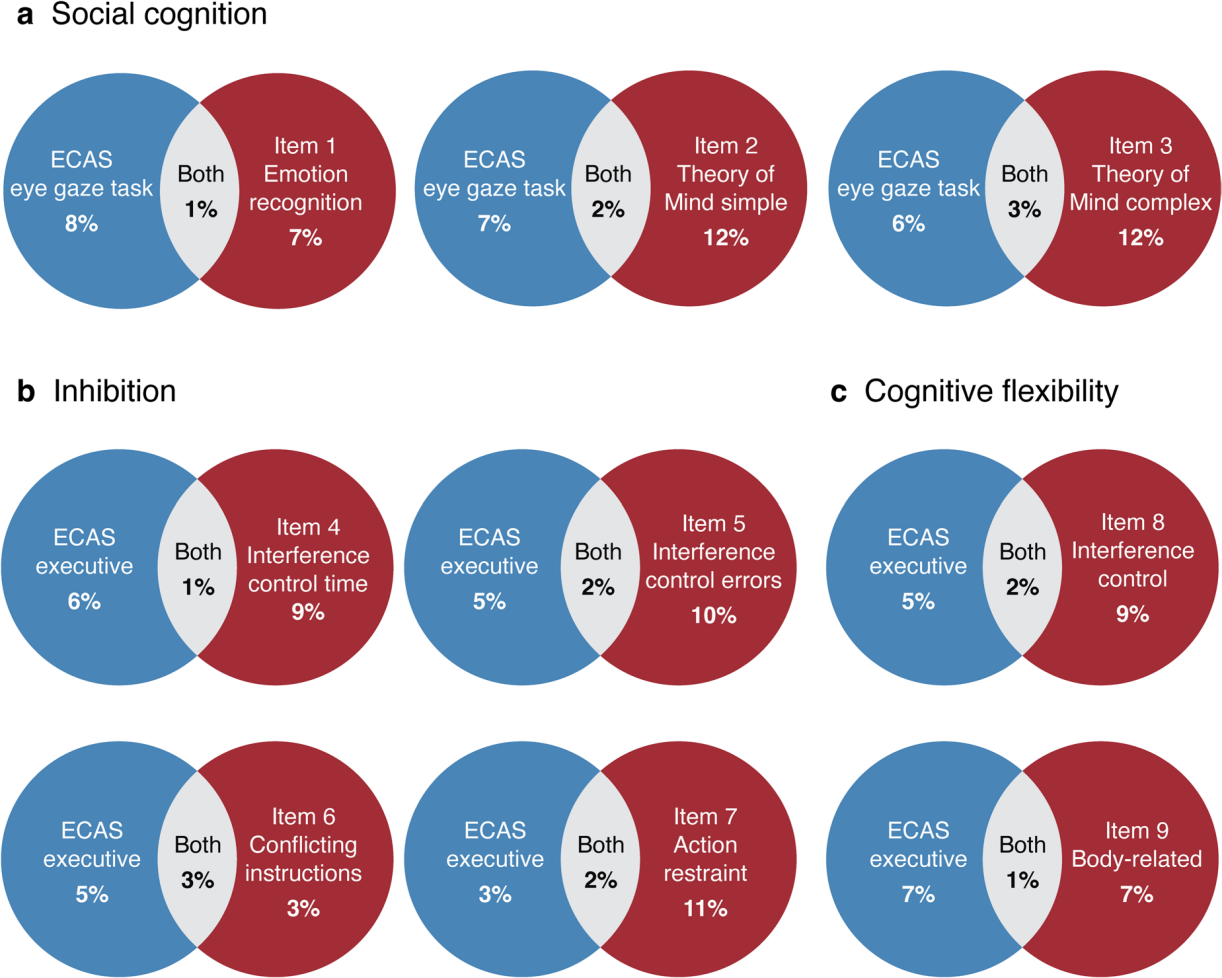
Comparison of impairments across domains affected within FTD spectrum Venn diagrams illustrating overlap of impairments per item within domains associated with the FTD spectrum: **(a)** comparison between ECAS eye-gaze task and C-CAS social cognition items, **(b)** comparison between ECAS executive function and C-CAS inhibition, and **(c)** comparison between ECAS executive function and C-CAS cognitive flexibility. The percentages represent the proportion of patients with impaired scores. To enable direct comparison, we established normative data and a cut-off score for the ECAS eye-gaze task, mirroring our approach with the C-CAS items, using control participant data. Due to missing data, there may be slight variations in percentages compared to those in Tables 1 and 2. *ECAS* Edinburgh Cognitive and Behavioural ALS Screen *C-CAS* Complementary Cognitive ALS Screen

**Fig. 4:**
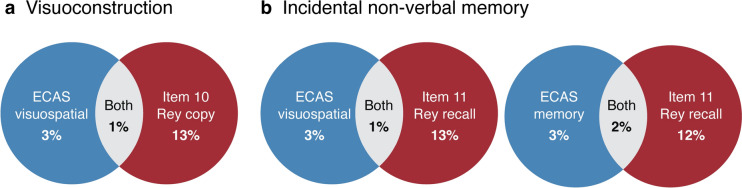
Comparison of impairments across domains affected beyond FTD spectrum Venn diagrams illustrating overlap of impairments per item within domains beyond the FTD spectrum: **(a)** comparing ECAS visuospatial function and C-CAS visuoconstruction, and **(b)** comparing ECAS visuospatial function and memory and C-CAS incidental non-verbal memory. The percentages represent the proportion of patients with impaired scores. Due to the inability to directly associate body orientation with a relevant ECAS domain, no comparison is available for this domain. Due to missing data, there may be slight variations in percentages compared to those in Tables 1 and 2. Abbreviations: *ECAS* Edinburgh Cognitive and Behavioural ALS Screen *C-CAS* Complementary Cognitive ALS Screen

When comparing impairments across domains affected within the FTD spectrum, the C-CAS detected cognitive impairments in social cognition in up to 12%, in executive function—inhibition in up to 11%, and in executive function—cognitive flexibility in up to 9% of the ALS patients that would not have been detected by the ECAS (Fig. 3). The overlap between impairments identified by both instruments remained low, ranging between 1 and 3%. When comparing impairments across domains beyond the FTD spectrum, the C-CAS found 12% more ALS patients with impaired visuoconstruction and 12–13% more with impaired incidental non-verbal memory, compared to those detected by the ECAS visuospatial and memory domain scores (Fig. 4).

#### Sensitivity analysis

There was no statistically significant association observed between anxiety or depression and the C-CAS sum score.

### C-CAS implementation

A free C-CAS web application has been developed. It requires only raw item scores and core clinical variables (age, sex, and education level) to calculate item-specific normal/impaired classifications based on the described normative data and the C-CAS sum score, which is a representation of overall cognitive performance within the C-CAS (https://apps4mnd.com/ccas/).

## Discussion

This study introduces the C-CAS: a novel, comprehensive, and brief multi-domain cognitive screening tool for ALS, designed to complement the ECAS. Through a cross-sectional, prospective case–control study involving 314 controls and 184 ALS patients, we identified impairments in cognitive functions not detected by previous screening tools. The C-CAS, a feasible and well-tolerated instrument, reveals impairments in social cognition, inhibition, cognitive flexibility, visuoconstruction, incidental non-verbal memory and body orientation, with 24% of patients exhibiting abnormal sum scores. Complementing the ECAS, the C-CAS detects impairments in 15% of patients that were not detected by the ECAS. The practical online tool provides C-CAS item-specific scores and sum score results, and classifies them as normal or impaired based on normative data collected in controls. Combining the C-CAS and ECAS in the cognitive screening of ALS can improve the recognition of individuals with and without cognitive impairments, enhancing both patient care and the development of targeted interventions.

Within the FTD spectrum, the C-CAS enhances the screening of social cognition and executive function, revealing impairments undetected by previous screening instruments. Our study identified impairments in emotion recognition and theory of mind, affecting up to 16% of the patients, even when the ECAS eye-gaze task showed no impairments. This illustrates that the C-CAS better captures the broadness of social cognition in its different components compared to the ECAS. Moreover, evaluating social cognition and executive function as separate domains in the C-CAS allows for a more precise assessment of these domains. Our study also revealed abnormalities in inhibition and cognitive flexibility (both subdomains of executive function) in up to 13% of ALS patients, largely not identified by the ECAS executive domain score. This suggests that items included in the C-CAS study complementary aspects of executive function compared to those within the ECAS. A possible explanation could be the increased use of non-verbal tasks assessing executive function, which may be particularly relevant for ALS patients with language deficits, or the incorporation of assessments focused on interference control. Language assessment, crucial for the Strong classification, is not included in the C-CAS due to the comprehensive evaluation provided by the ECAS [2]. Impairments in emotion recognition, theory of mind, inhibition and cognitive flexibility align with results from comprehensive neuropsychological testing in ALS [3, 5, 24, 25]. Uncovering impairments in social cognition, such as emotion recognition or theory of mind, and impulsivity and switching difficulties in ALS through cognitive screening can offer valuable insights into patients’ adaptive capacity. This information is essential to optimize communication, education and support, allowing for tailored counseling for both patients and caregivers.

The C-CAS enables systematic and efficient detection of cognitive functions extending beyond the FTD spectrum. Our study identified cognitive impairments in visuoconstruction, incidental non-verbal memory and body orientation, affecting around 14% of ALS patients. There was minimal overlap (1–2%) between visuoconstruction and ECAS visuospatial impairments. This could be attributed to the related but distinct nature of these functions. In the ECAS visuospatial domain, tasks primarily focus on visuoperception, whereas the C-CAS also examines construction abilities [26]. In addition, the C-CAS investigates non-verbal memory, as opposed to verbal memory in the ECAS, resulting in minimal overlap (1–2%) between impairments in our study. Previous neuropsychological research reported deficits in non-verbal memory and visuoconstruction, but until now, systematic screening for these in the cognitive assessment of ALS was not possible [3, 5, 27]. There is ongoing debate on whether memory and visuoconstructive impairments stem from executive dysfunction. The low correlation between executive and memory items in the C-CAS may indicate that impairments in these domains are not solely a consequence of executive dysfunction. Impairments in posterior brain functions, such as body orientation, have been found previously, although only limited studies investigated this [5]. Our findings align with the recent identification of a new neuroimaging phenotype characterized by involvement of posterior cingulate, parietal, temporal and cerebellar regions [8]. In addition, a case report of cortical posterior atrophy in ALS demonstrates that posterior brain function impairments can occur in ALS [28]. Recognizing deficits outside the FTD spectrum could be essential, as they might require distinct guidance and care compared to frontotemporal problems. Our findings may challenge the idea that cognitive deficits in ALS exclusively align with a range of FTD-like changes. As we did not clearly find more deficits in domains commonly affected within rather than beyond the FTD spectrum, this may suggest that these domains could be integral to the ALS cognitive profile. In contrast to previous screening tools, the C-CAS allows us to screen systematically for deficits extending beyond the FTD spectrum, providing deeper insights into the heterogeneity of cognitive dysfunction in ALS.

Combining the C-CAS with the ECAS allows us to screen for a broad spectrum of cognitive deficits and assess a larger part of the heterogeneity, holding promise for advancing both research and clinical care. Given the practical challenges of conducting neuropsychological investigations for all ALS patients, screening instruments offer a concise yet comprehensive means of gathering valuable information about cognition. Rather than relying on ECAS alone, if normal scores are observed on both the ECAS and C-CAS, we can be more confident that cognitive problems are not present. In our study, normal scores on both cognitive screens were observed in 73% of the patients (65% when also considering the absence of behavioral changes). By integrating the C-CAS with the ECAS, we gain insight into cognitive heterogeneity, facilitating the identification of existing and new ALS subtypes. Low domain scores on the C-CAS, for example in social cognition, may identify deficits relevant to the Strong criteria [2]. Furthermore, the C-CAS screens for cognitive deficits beyond the FTD spectrum. Taken together, and in correlation with clinical findings, this screener assists clinicians in identifying cognitive deficits and considering further neuropsychological assessment. Improved recognition of cognitive phenotypes in ALS, whether normal or impaired, is crucial for personalized counseling of patients and in guiding treatment decisions, directly impacting their daily lives [3]. Also, this is important in clinical trials, especially when cognitive impairments are determining factors for inclusion or exclusion, or when stratification is based on subgroups [6]. To gain a better understanding of the development and trajectory of cognitive problems, longitudinal research is needed, applying parallel versions of the C-CAS. Moreover, since various cognitive impairments have different patterns of underlying cerebral degeneration, the C-CAS may help reveal these neurodegenerative patterns and possibly their mechanisms. This could be a step towards developing targeted therapies to slow or prevent cognitive deterioration in ALS.

FTD, which is at one end of the ALS-FTD spectrum, might benefit from the C-CAS to identify signs of cognitive dysfunction. FTD is characterized by changes in behavior, personality, emotion and executive control [29]. Diagnosis requires the presence of specific criteria, such as disinhibition, apathy, lack of empathy, compulsions, hyperorality, and executive dysfunction [30]. These criteria are predominantly behavioral, but also encompass cognitive changes. Since the C-CAS includes items that directly assess key FTD domains like inhibition, cognitive flexibility, and social cognition, the screen may provide a quick test for obtaining insight into cognitive changes that can occur in FTD and provide a standardized measure for the severity of these changes.

We acknowledge the limitations of our study. Our study population consists of ALS patients in the early stages of the disease who exhibit a relatively high level of functioning. However, this early stage represents a critical period for evaluation, as early recognition of cognitive phenotypes could facilitate personalized counseling and enhance patient eligibility for clinical trials. As the disease progresses, patients may find certain tasks increasingly challenging due to motor disabilities. While 93% of the participants were able to complete all items and a sum score could still be calculated even if certain items were missing (see Methods section), severe motor problems precluded administration of some items in a small percentage of patients. Another limitation is the absence of important characteristics like *C9orf72*-status and classifications according to the Strong criteria (FTD, ALS-ci, ALS-bi, and ALS-cbi), in some of the patients. We used these factors mainly as a description of the population studied; exploring the relationship between the C-CAS and demographic characteristics was not within the scope of this study. Finally, in future research, it is important to compare the screen against a comprehensive neuropsychological assessment for validation purposes. The inclusion of a large control group in our study enhances the precision and reliability of our results, but comparison with a comprehensive neuropsychological assessment might offer more detailed information regarding the precise interpretation of individual items.

In conclusion, the C-CAS is a systematic and comprehensive screener of a broad spectrum of cognitive functions in ALS, complementing the ECAS. Our study identified cognitive deficits both within and beyond the FTD spectrum, which might challenge the notion that cognitive deficits in ALS exclusively align with a spectrum of FTD-like changes. Evaluated in a large cohort of patients and controls, our study revealed normal scores on both the C-CAS and ECAS in 73% of ALS patients (65% when considering the absence of behavioral changes). Among the 27% with cognitive impairments, 5% were exclusively identified by the ECAS, and 15% were exclusively identified by the C-CAS. Our ALS-tailored screen is well-tolerated and brief to administer (15–20 min), making it clinically feasible to integrate into routine care, further enhanced by the practical online scoring tool that includes normative data. By employing the C-CAS alongside the ECAS, we can systematically screen a larger portion of the cognitive heterogeneity in ALS, enabling the identification of different ALS phenotypes. This standardized approach enhances personalized patient care and contributes to stratification in clinical trials and the development of targeted interventions.

## Supplementary Information

Below is the link to the electronic supplementary material.

Supplementary file1 (DOCX 3799 KB)

Supplementary file2 (TIF 5112 KB)

Supplementary file3 (PDF 2493 KB)

Supplementary file4 (PDF 2001 KB)

Supplementary file5 (PDF 1592 KB)

Supplementary file6 (PDF 2296 KB)

## Data Availability

The data supporting this study’s findings are available from the corresponding author upon reasonable request.
