# Supplementary material for: Cognitive impairment within and beyond the FTD spectrum in ALS: development of a complementary cognitive screen
Source: J Neurol. 2025 Mar 13;272(4):268. doi: 10.1007/s00415-025-13006-2 (PMC11903523; doi:10.1007/s00415-025-13006-2)
Supplement: Supplementary file 1 — Supplementary file1 (DOCX 3799 KB) [file 415_2025_13006_MOESM1_ESM.docx]

**Journal of Neurology - Original communication**

**Cognitive impairment within and beyond the FTD spectrum in ALS: development of a complementary cognitive screen**

Annebelle Michielsen, MD, Kevin van Veenhuijzen, MD, Fenna Hiemstra, Ilse M. Jansen, Boaz Kalkhoven, Jan H. Veldink, MD, PhD, Esther T. Kruitwagen, MD, PhD, Michael van Es, MD, PhD, Martine J. E. van Zandvoort, PhD, Leonard H. van den Berg, MD, PhD**^†^**, Henk-Jan Westeneng, MD, PhD**^†^**

^†^These authors contributed equally to this work.

Corresponding author: Henk-Jan Westeneng, MD, PhD.

Corresponding author’s affiliation: Department of Neurology, UMC Utrecht Brain Center, University Medical Center Utrecht, 3508 GA Utrecht, the Netherlands

Corresponding author’s e-mail address: [H.J.Westeneng@umcutrecht.nl](mailto:H.J.Westeneng@umcutrecht.nl)

**Supplementary material**

**Supplementary Table 1** Regression characteristics per item of C-CAS

| C-CAS item | Scale | Model | Covariables |
| --- | --- | --- | --- |
| Item 1 – Emotion recognition | Count | Poisson regression | Age |
| Item 2 – Theory of Mind simple | Count | Poisson regression | Age |
| Item 3 – Theory of Mind complex | Count | Negative binomial regression | Age, sex |
| Item 4 – Interference control time | Continuous | Shifted log-normal regression | Age, education |
| Item 5 – Interference control errors | Count | Negative binomial regression | Age, education |
| Item 6 – Conflicting instructions | Ordinal | Ordinal logistic regression | Education |
| Item 7 – Action restraint | Ordinal | Ordinal logistic regression | Age |
| Item 8 – Cognitive flexibility interference control | Count | Negative binomial regression | Age, education |
| Item 9 – Cognitive flexibility body-related | Count | Negative binomial regression | Education |
| Item 10 – Rey copy | Count | Negative binomial regression | Age, education |
| Item 11 – Rey recall | Count | Negative binomial regression | Age, sex, education |
| Item 12 – Body orientation total | Count | Negative binomial regression | Age, education |

Data and regression characteristics per C-CAS item. Items are displayed in a hierarchical order, with those within the FTD spectrum appearing before those beyond the FTD spectrum.

**Supplementary Table 2** Normative cut-off scores for residuals per item of C-CAS

| C-CAS | Cut-off |
| --- | --- |
| Item 1 – Emotion recognition | -0.80 |
| Item 2 – Theory of Mind simple | -0.83 |
| Item 3 – Theory of Mind complex | -1.37 |
| Item 4 – Interference control time | -0.77 |
| Item 5 – Interference control errors | -0.67 |
| Item 6 – Conflicting instructions | 0.13 |
| Item 7 – Action restraint | -0.58 |
| Item 8 – Cognitive flexibility interference control | -1.06 |
| Item 9 – Cognitive flexibility body related | -1.22 |
| Item 10 – Rey copy | -1.46 |
| Item 11 – Rey recall | -1.50 |
| Item 12 – Body orientation total | -1.34 |
| C-CAS Sum score | -8.34 |

Cut-off of residuals corresponding to the lowest 5^th^ percentile rank in controls. Items are displayed in a hierarchical order, with those within the FTD spectrum appearing before those beyond the FTD spectrum.


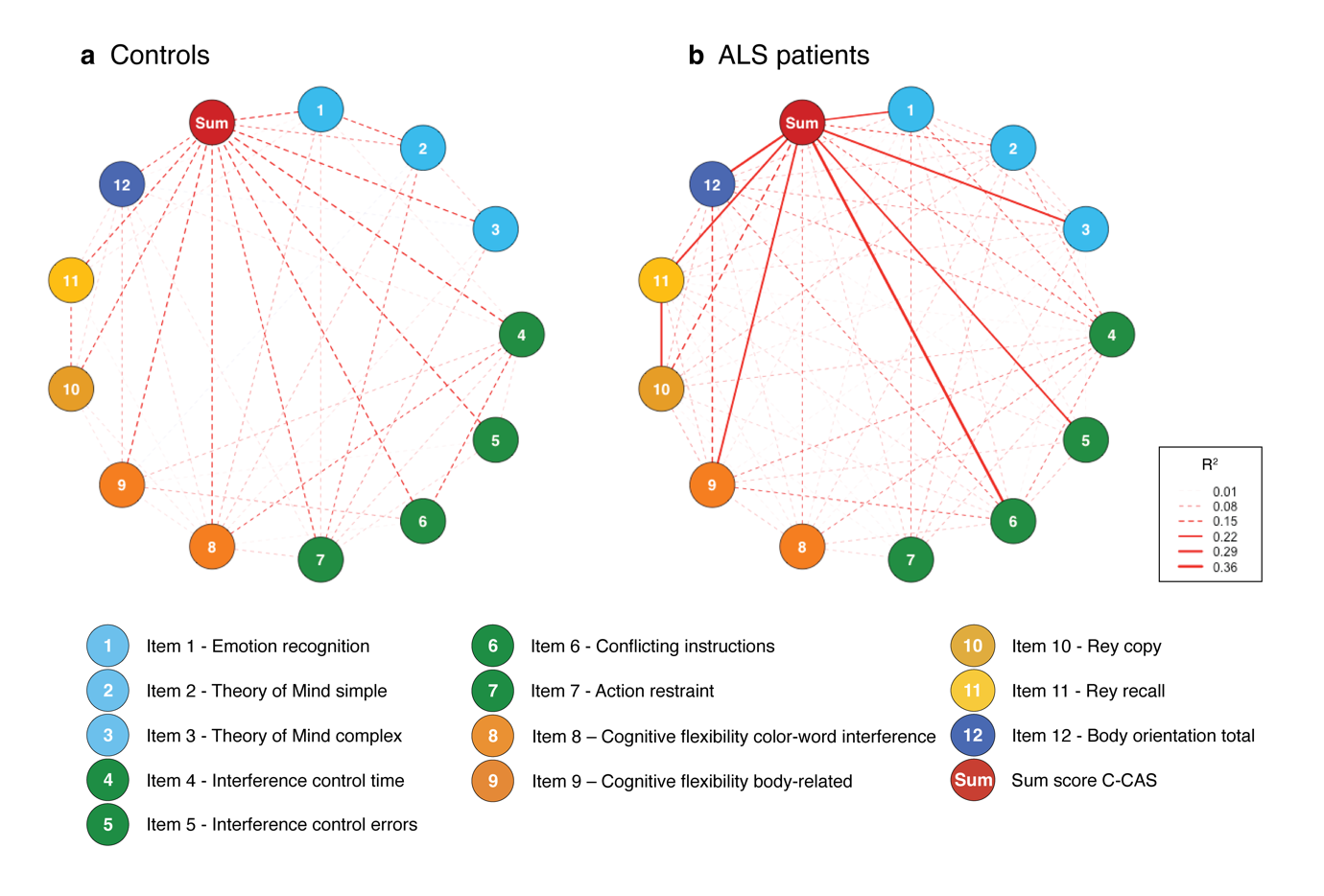


**Supplementary Fig. 1** Pairwise correlations between individual items of C-CAS Pairwise correlations between individual items of the C-CAS for controls **(a)** and ALS patients **(b)**. Edges indicate the correlation coefficient (R^2^) between items. Items sharing the same color belong to the same domains. Light blue for social cognition, green for inhibition, orange for cognitive flexibility, gold for visuoconstruction, bright yellow for incidental non-verbal memory, and dark blue for body orientation.
