# Supplementary figures and images for: Cognitive impairment within and beyond the FTD spectrum in ALS: development of a complementary cognitive screen
Source: J Neurol. 2025 Mar 13;272(4):268. doi: 10.1007/s00415-025-13006-2 (PMC11903523; doi:10.1007/s00415-025-13006-2)

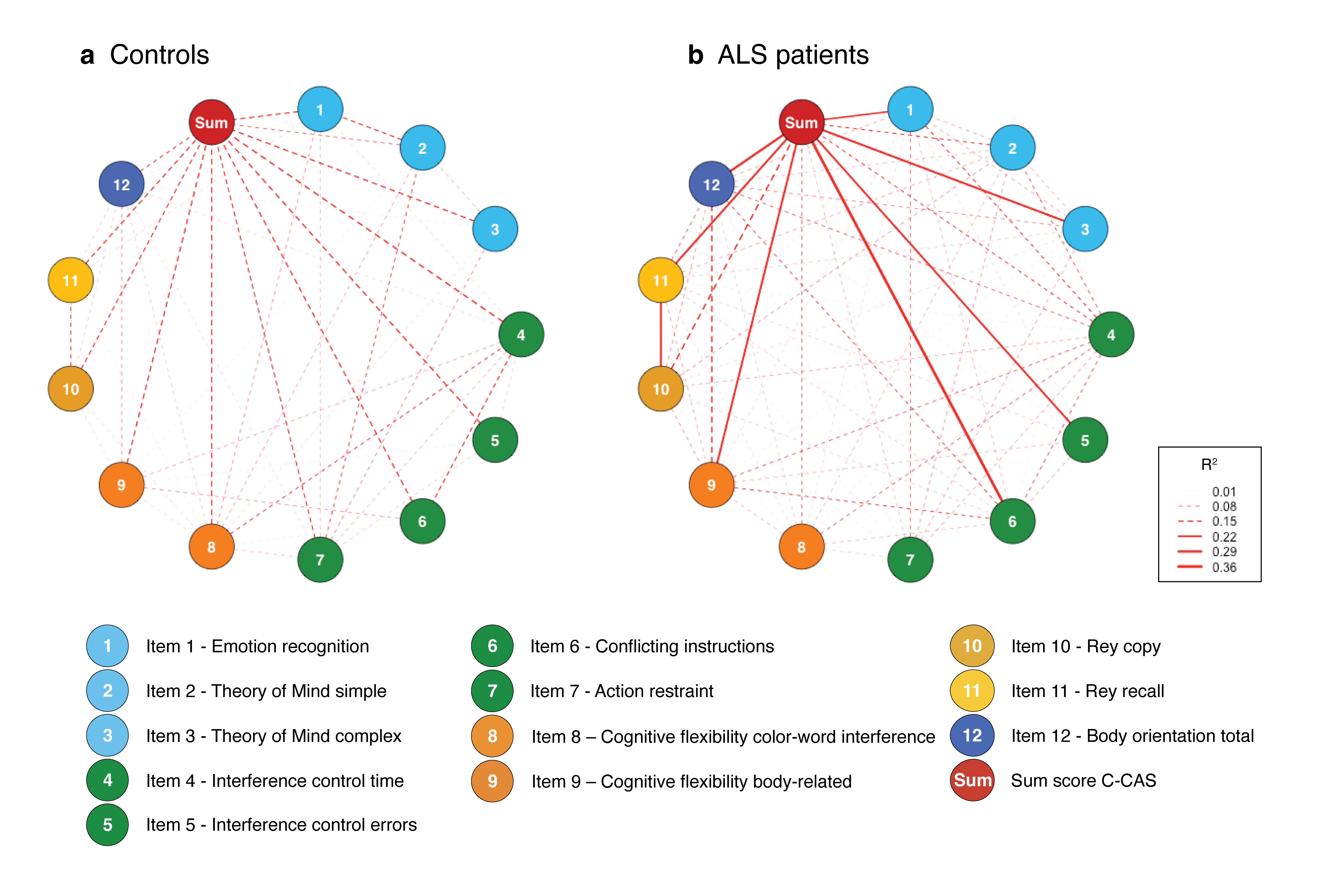

Supplement: Supplementary file 2 — Supplementary file2 (TIF 5112 KB) [file 415_2025_13006_MOESM2_ESM.tif]
