# Supplementary material for: Cognitive impairment within and beyond the FTD spectrum in ALS: development of a complementary cognitive screen
Source: J Neurol. 2025 Mar 13;272(4):268. doi: 10.1007/s00415-025-13006-2 (PMC11903523; doi:10.1007/s00415-025-13006-2)
Supplement: Supplementary file 3 — Supplementary file3 (PDF 2493 KB) [file 415_2025_13006_MOESM3_ESM.pdf]

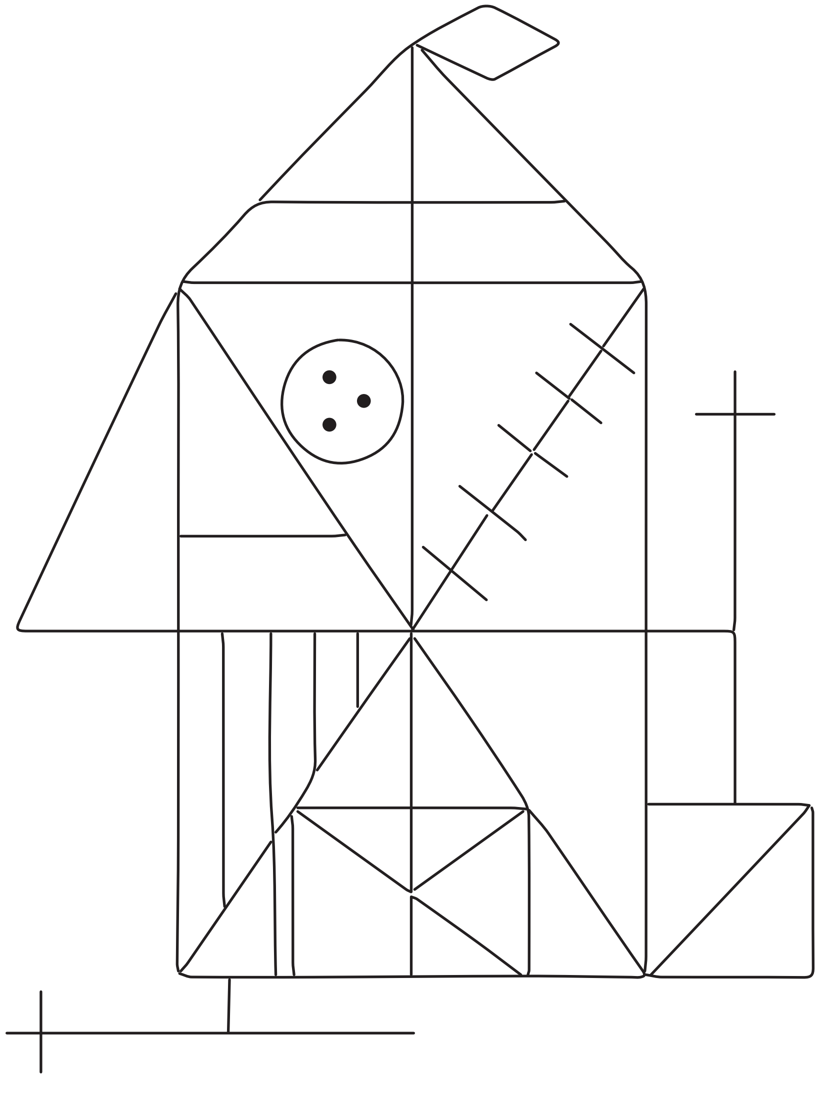

© Copyright, the University Medical Center Utrecht, 2024. All Rights Reserved.

Practice round task 2 (part I)

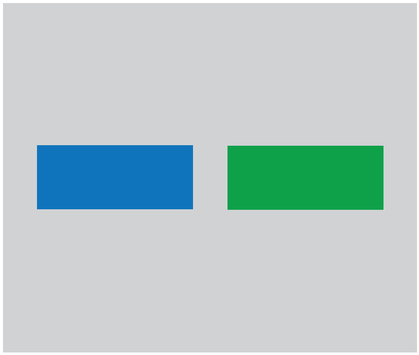

© Copyright, the University Medical Center Utrecht, 2024. All Rights Reserved.

Task 2 (part I)

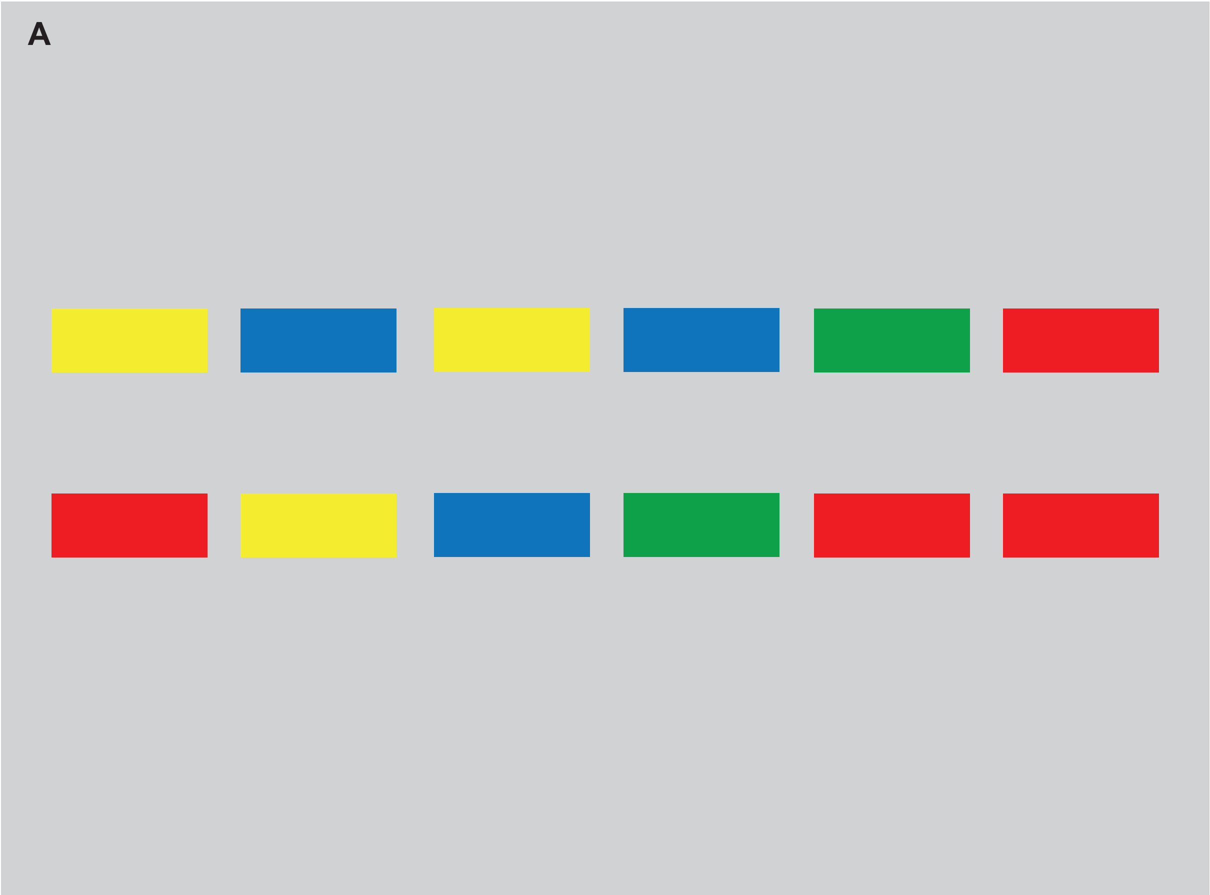

© Copyright, the University Medical Center Utrecht, 2024. All Rights Reserved.

Task 2 (part I)

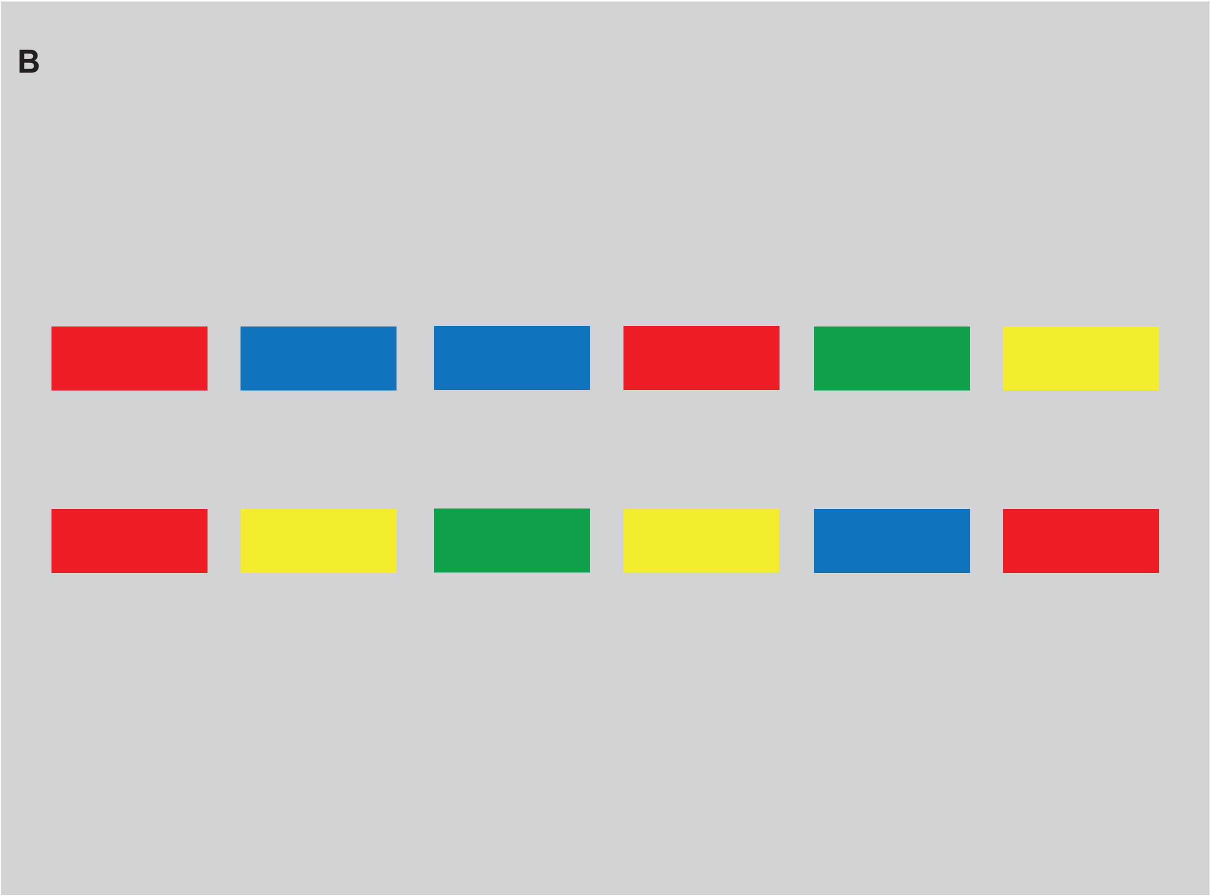

© Copyright, the University Medical Center Utrecht, 2024. All Rights Reserved.

Practice round task 3 (part II)

blue **yellow**

© Copyright, the University Medical Center Utrecht, 2024. All Rights Reserved.

Task 3 (part II)

A

yellow	yellow	yellow	green	green	green
red	green	green	yellow	red	red

© Copyright, the University Medical Center Utrecht, 2024. All Rights Reserved.

Task 3 (part II)

B

red	red	yellow	green	green	blue
green	blue	blue	yellow	blue	green

© Copyright, the University Medical Center Utrecht, 2024. All Rights Reserved.

Practice round task 4 (part III)

yellow green

© Copyright, the University Medical Center Utrecht, 2024. All Rights Reserved.

Task 4 (part III)

A

red	green	green	red	yellow	yellow
green	yellow	green	green	red	green

© Copyright, the University Medical Center Utrecht, 2024. All Rights Reserved.

Task 4 (part III)

B

red	yellow	red	red	yellow	green
green	blue	green	red	green	red

© Copyright, the University Medical Center Utrecht, 2024. All Rights Reserved.

Practice round task 5 (part IV)

red yellow

© Copyright, the University Medical Center Utrecht, 2024. All Rights Reserved.

Task 5 (part IV)

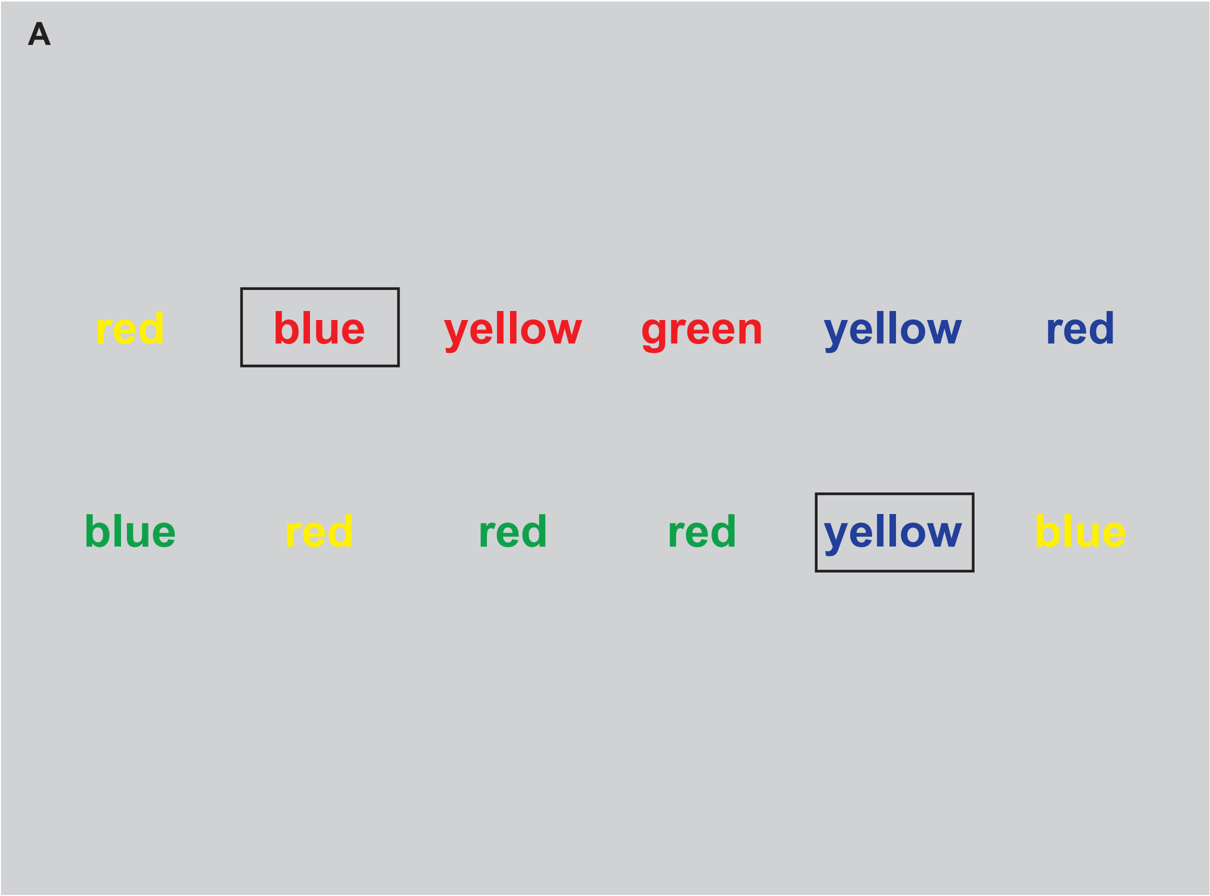

© Copyright, the University Medical Center Utrecht, 2024. All Rights Reserved.

Task 5 (part IV)

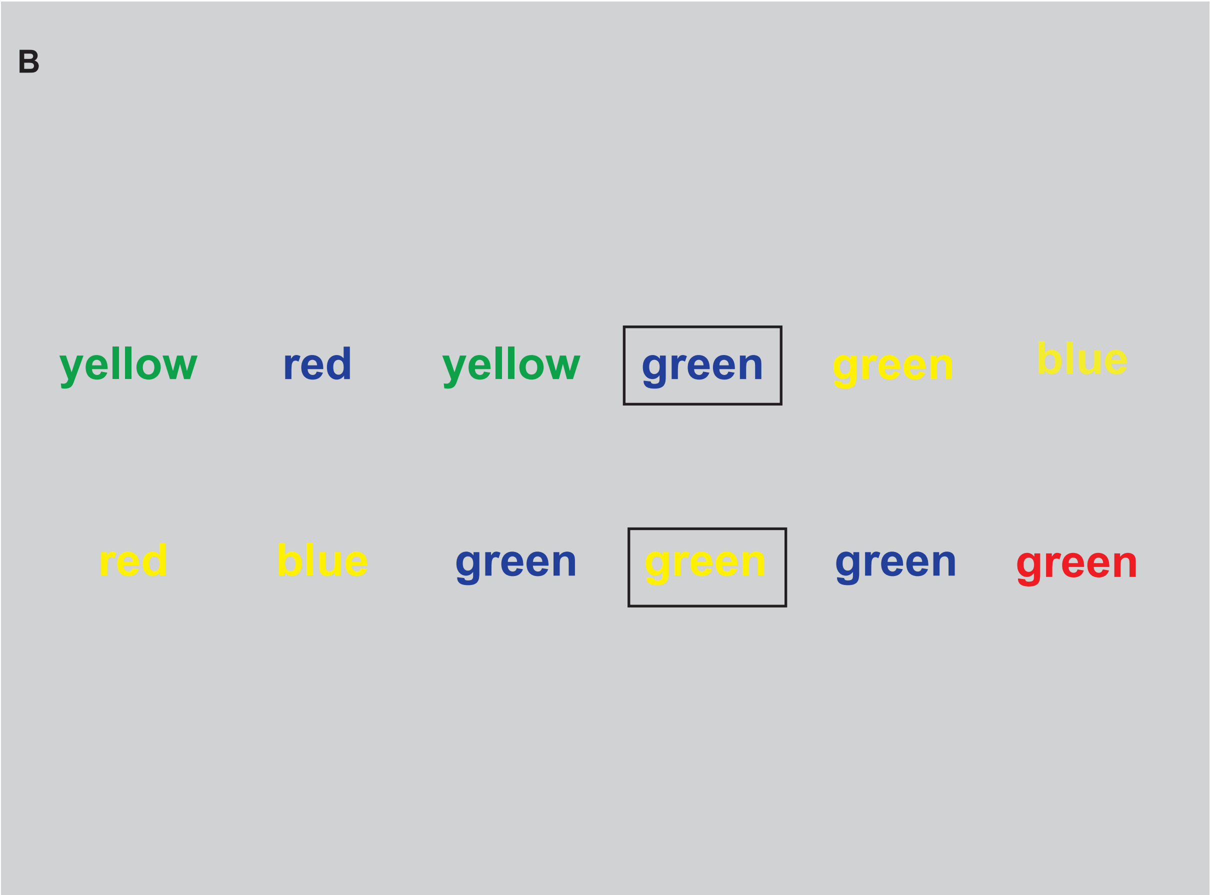

© Copyright, the University Medical Center Utrecht, 2024. All Rights Reserved.

Practice round task 6 (part I)

Spoken

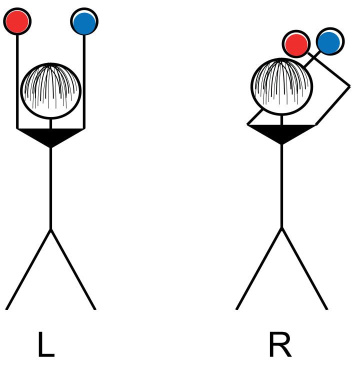

© Copyright, the University Medical Center Utrecht, 2024. All Rights Reserved.

Task 6 (part I)

Spoken

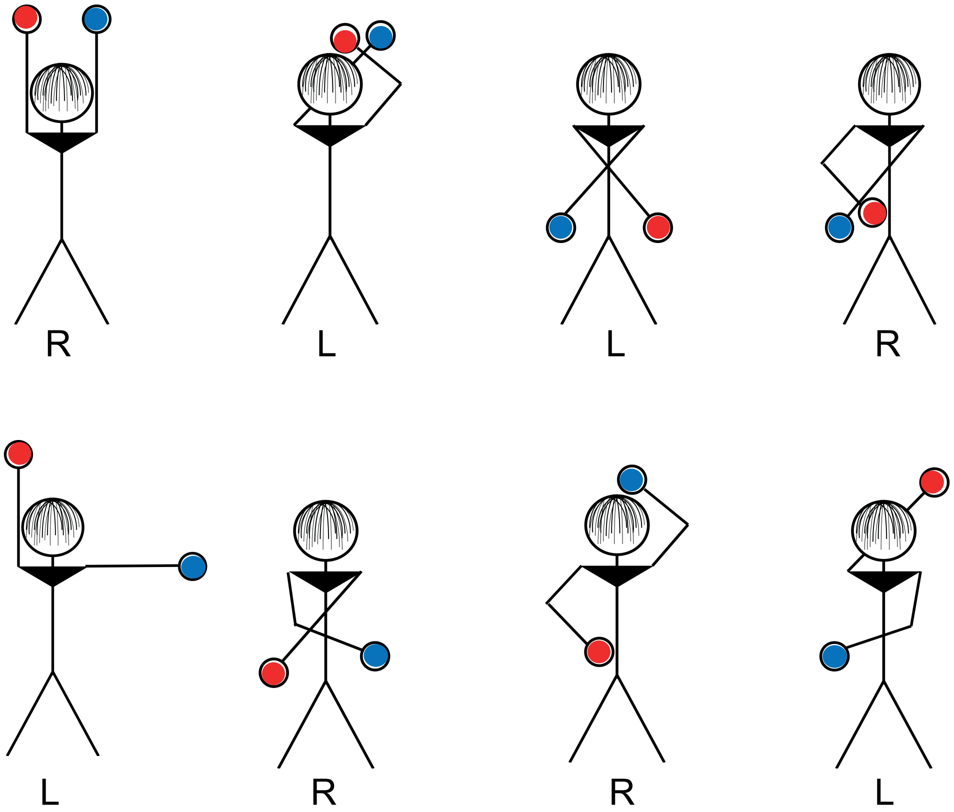

© Copyright, the University Medical Center Utrecht, 2024. All Rights Reserved.

Practice round task 7 (part II)

Spoken

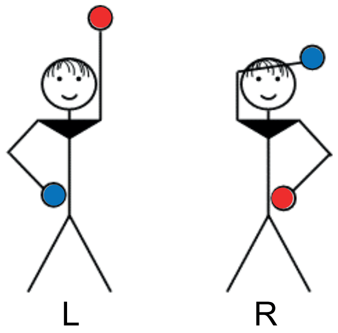

© Copyright, the University Medical Center Utrecht, 2024. All Rights Reserved.

Task 7 (part II)

Spoken

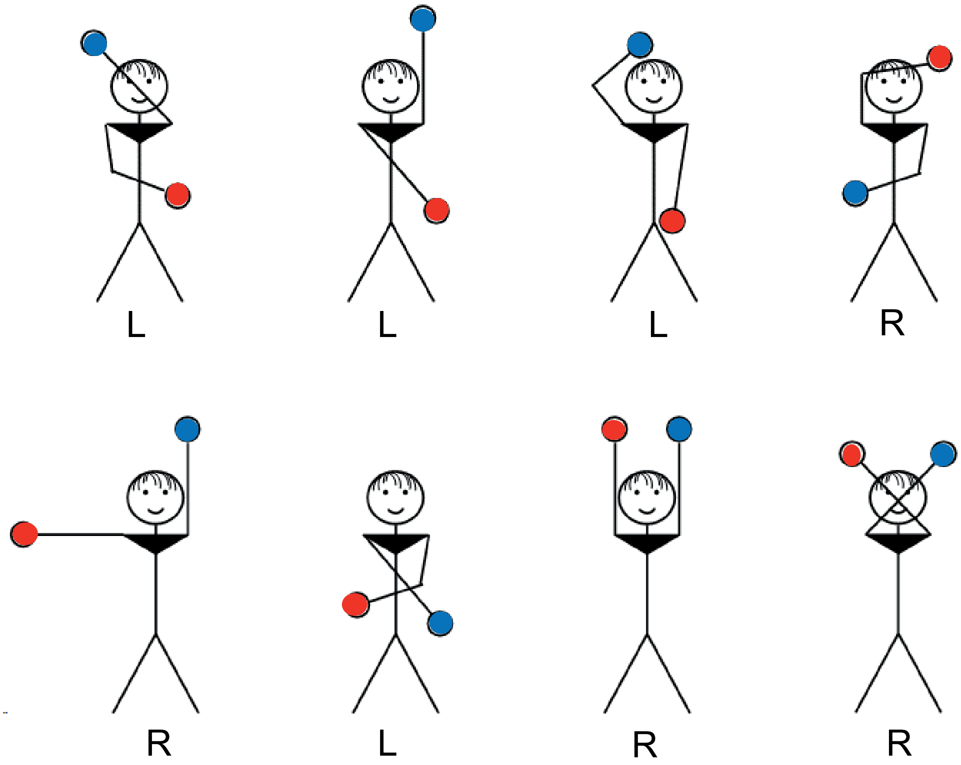

© Copyright, the University Medical Center Utrecht, 2024. All Rights Reserved.

Practice round task 8 (part III)

Spoken

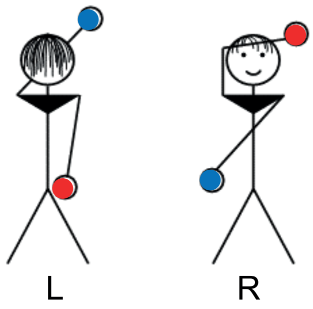

© Copyright, the University Medical Center Utrecht, 2024. All Rights Reserved.

Task 8 (part III)

Spoken

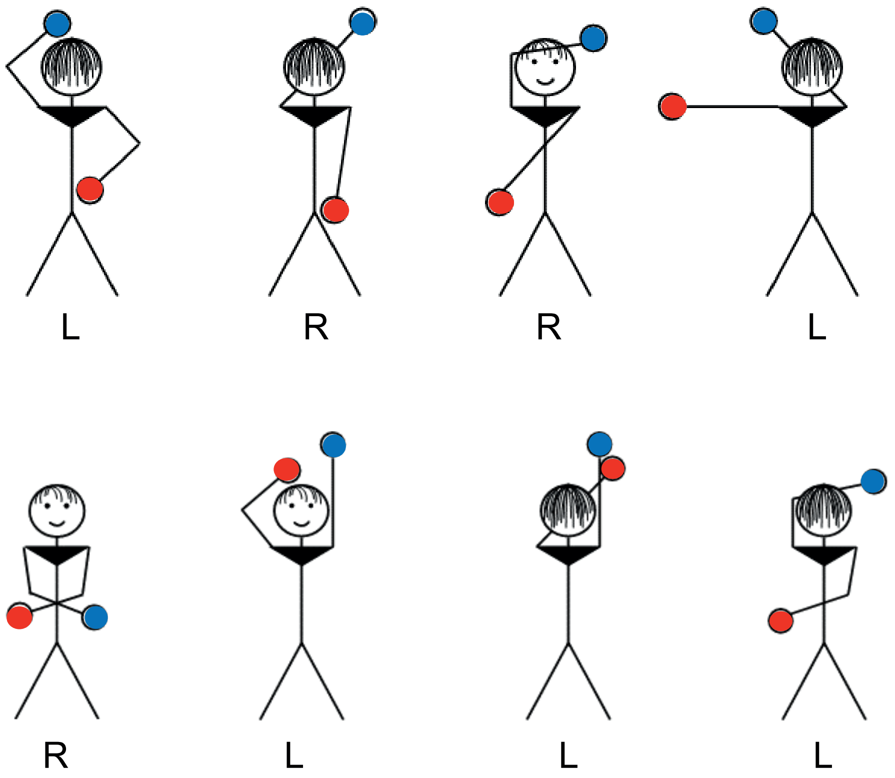

© Copyright, the University Medical Center Utrecht, 2024. All Rights Reserved.

Task 9
Spoken

1

1. HAPPY 2. SURPRISED
3. ANGRY 4. DISGUST

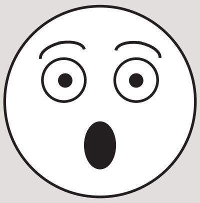

2

1. AFRAID 2. ANGRY
3. HAPPY 4. DISGUST

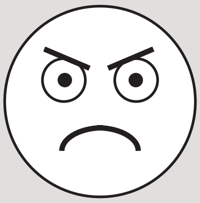

3

1. SAD 2. SURPRISED
3. ANGRY 4. AFRAID

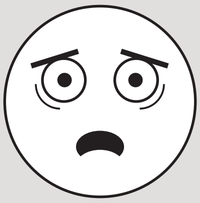

4

1. AFRAID 2. ANGRY
3. HAPPY 4. SAD

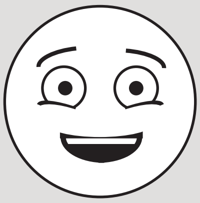

5

1. SAD 2. DISGUST
3. HAPPY 4. AFRAID

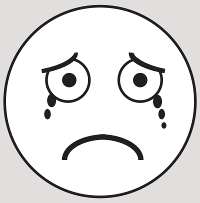

6

1. AFRAID 2. ANGRY
3. HAPPY 4. DISGUST

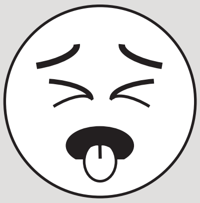

© Copyright, the University Medical Center Utrecht, 2024. All Rights Reserved.

Task 10
Spoken

Who thinks your joke is funny?

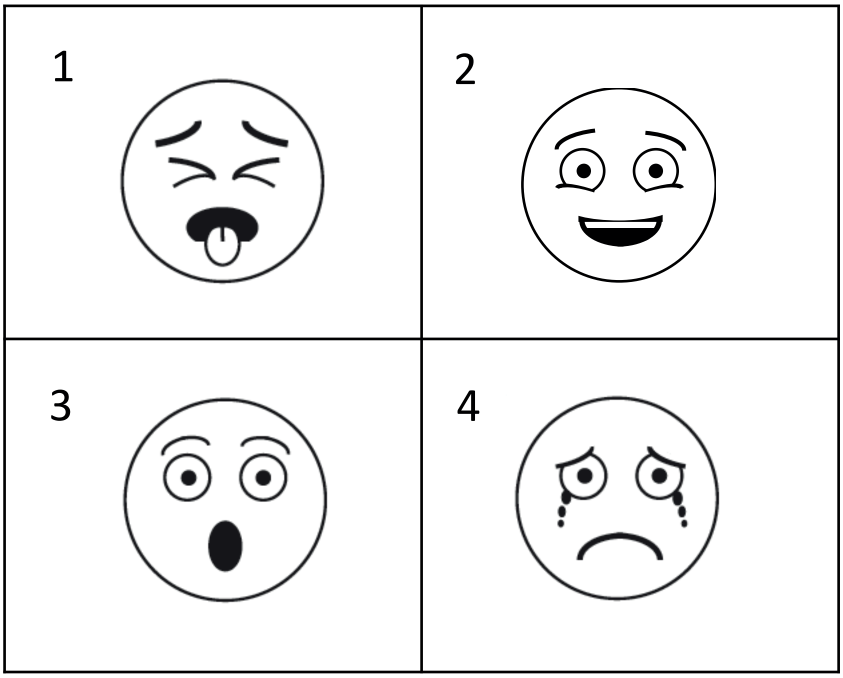

Who thinks your cake is not delicious?

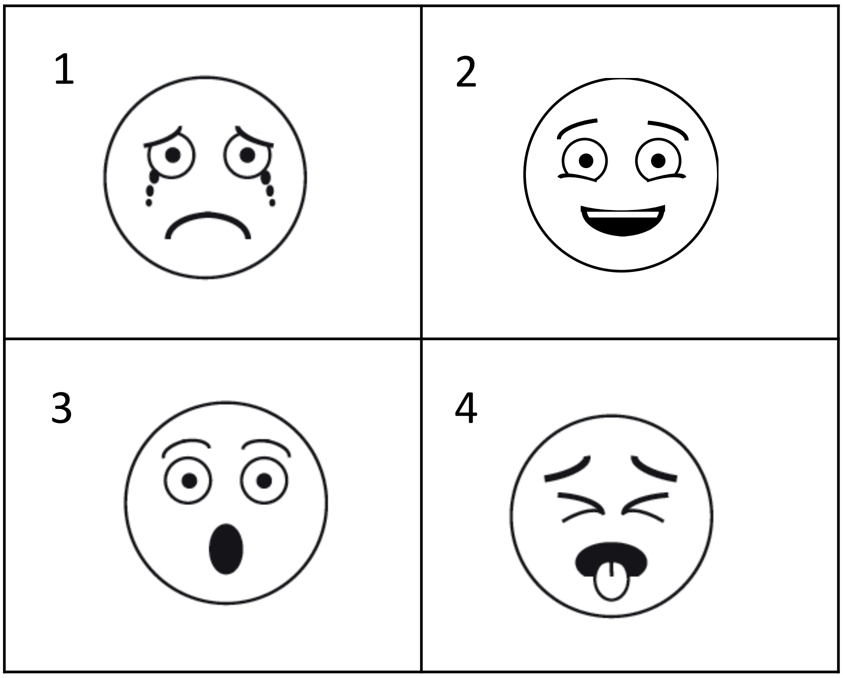

© Copyright, the University Medical Center Utrecht, 2024. All Rights Reserved.

Task 10
Spoken

Who needs your comfort?

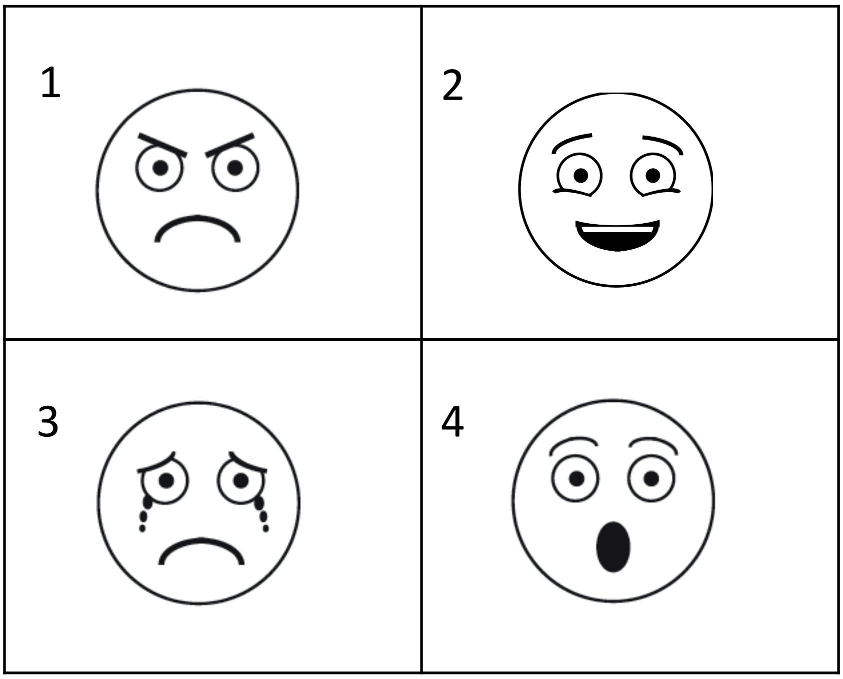

Who scares you?

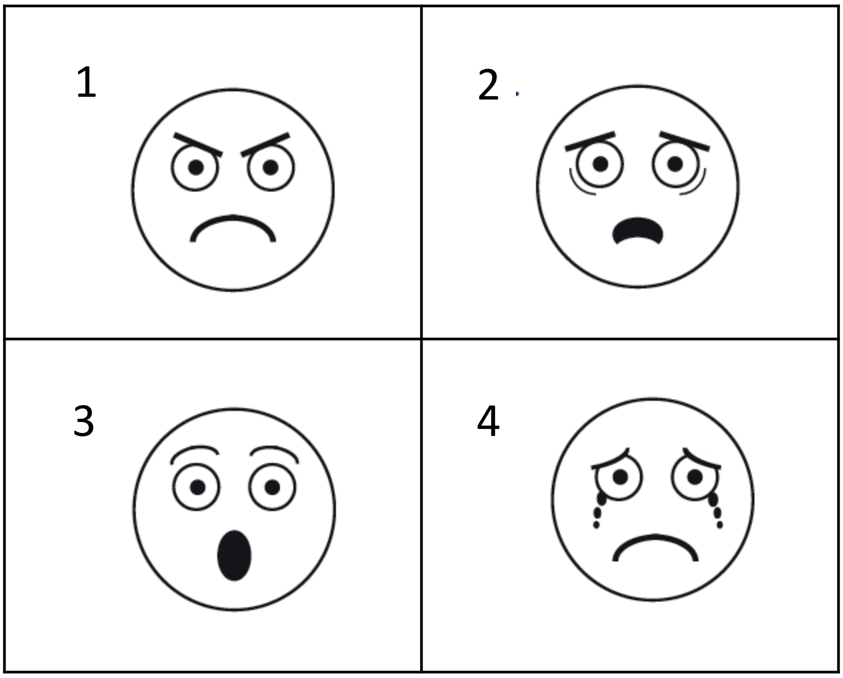

© Copyright, the University Medical Center Utrecht, 2024. All Rights Reserved.

Task 10
Spoken

Who did you just surprise?

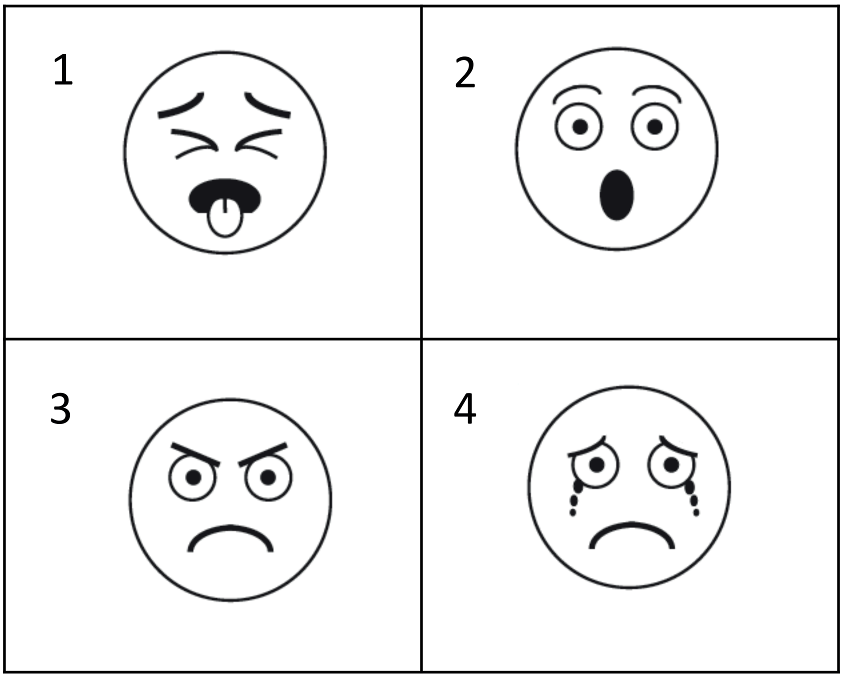

Who did you just show a scary film to?

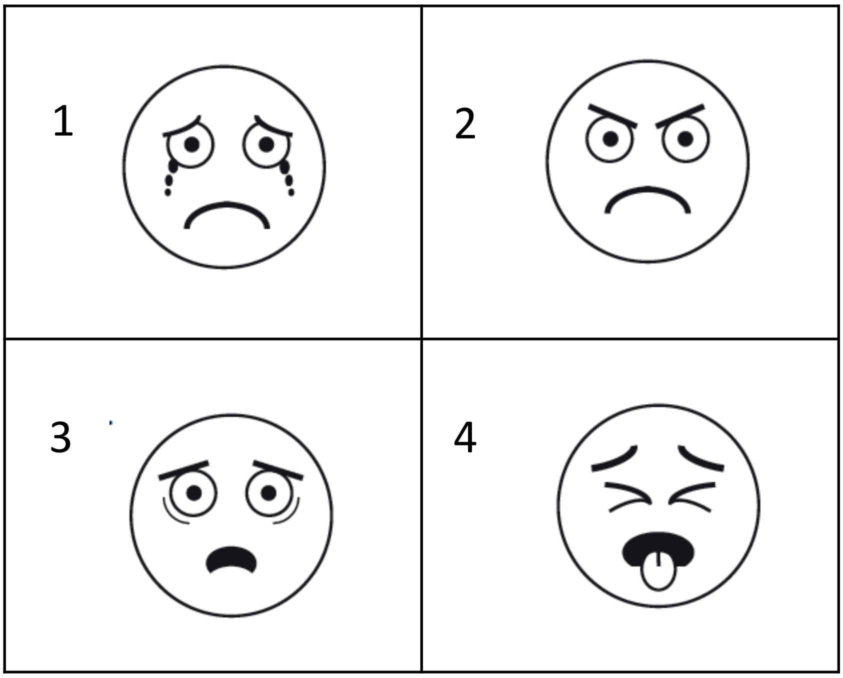

© Copyright, the University Medical Center Utrecht, 2024. All Rights Reserved.

Practice round task 6 (part I)

Written

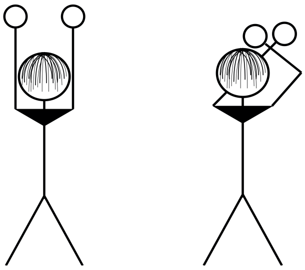

L R

© Copyright, the University Medical Center Utrecht, 2024. All Rights Reserved.

Practice round task 7 (part II)

Written

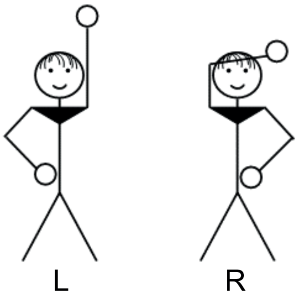

© Copyright, the University Medical Center Utrecht, 2024. All Rights Reserved.

Practice round task 8 (part III)

Written

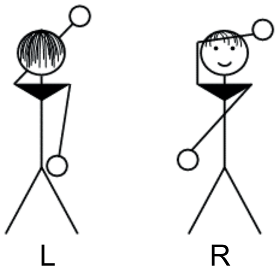

© Copyright, the University Medical Center Utrecht, 2024. All Rights Reserved.
