# Supplementary material for: Cognitive impairment within and beyond the FTD spectrum in ALS: development of a complementary cognitive screen
Source: J Neurol. 2025 Mar 13;272(4):268. doi: 10.1007/s00415-025-13006-2 (PMC11903523; doi:10.1007/s00415-025-13006-2)
Supplement: Supplementary file 4 — Supplementary file4 (PDF 2001 KB) [file 415_2025_13006_MOESM4_ESM.pdf]

## **Task 6: Body orientation - Part I** *Written*

Number:

Date:

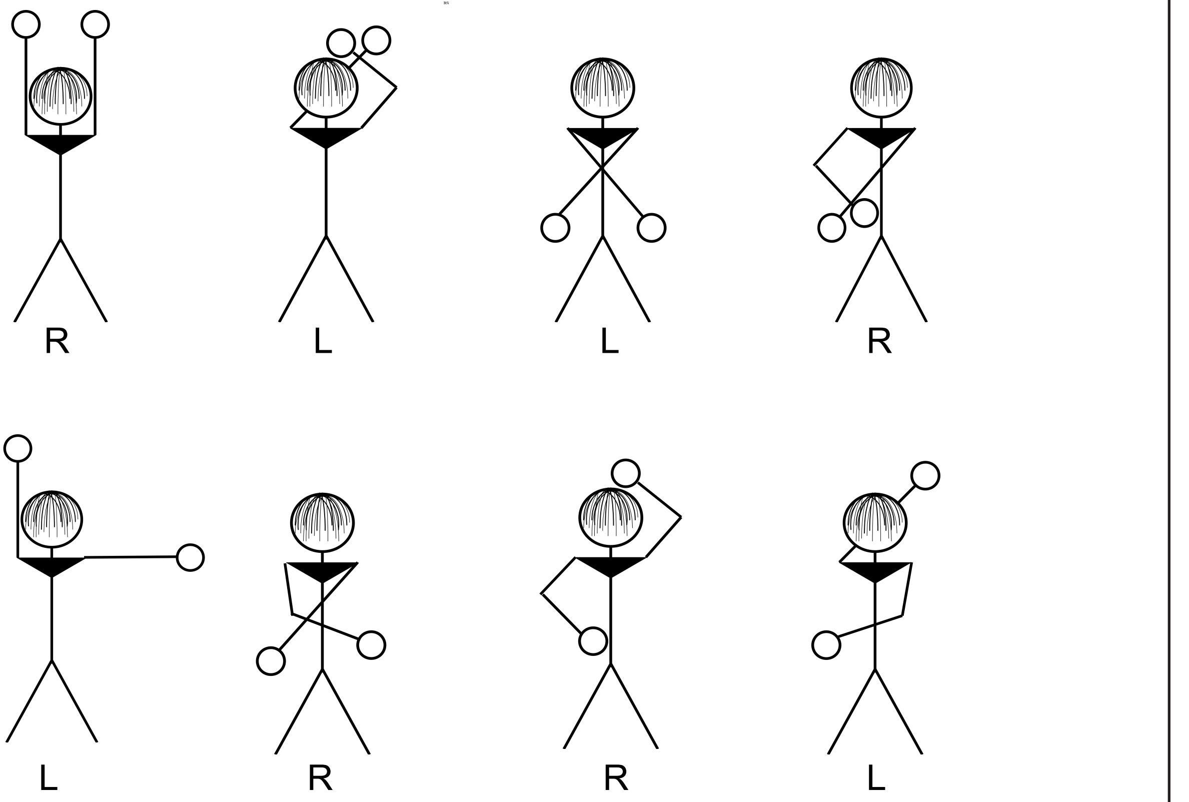

© Copyright, the University Medical Center Utrecht, 2024. All Rights Reserved.

## Task 7: Body orientation - Part II *Written*

Number:
Date:

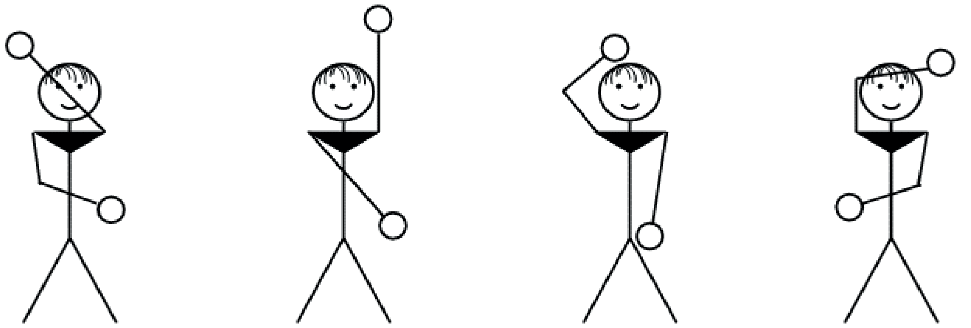

L L L R

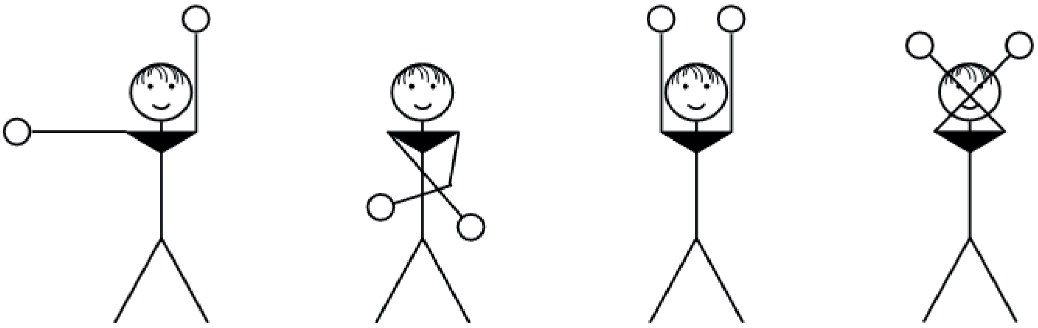

R L R R

© Copyright, the University Medical Center Utrecht, 2024. All Rights Reserved.

## Task 8: Body orientation - Part III *Written*

Number:
Date:

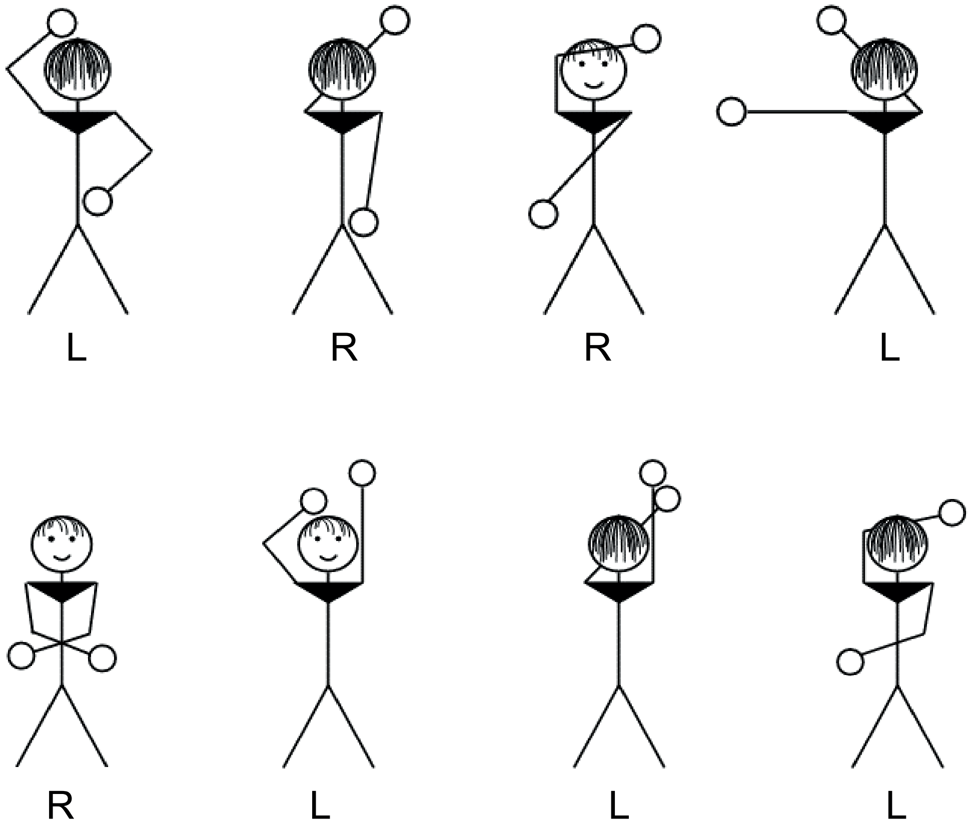

© Copyright, the University Medical Center Utrecht, 2024. All Rights Reserved.

## Task 9: Social cognition - Emotion recognition *Written*

Number:
Date:

**1**

1. HAPPY
2. SURPRISED
3. ANGRY
4. DISGUST

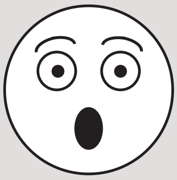

**2**

1. AFRAID
2. ANGRY
3. HAPPY
4. DISGUST

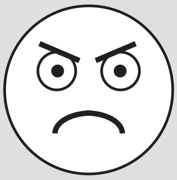

**3**

1. SAD
2. SURPRISED
3. ANGRY
4. AFRAID

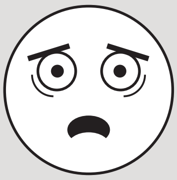

**4**

1. AFRAID
2. ANGRY
3. HAPPY
4. SAD

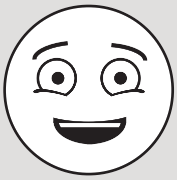

**5**

1. SAD
2. DISGUST
3. HAPPY
4. AFRAID

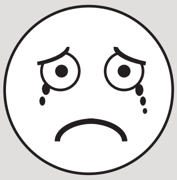

**6**

1. AFRAID
2. ANGRY
3. HAPPY
4. DISGUST

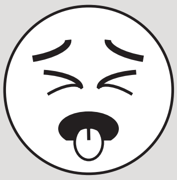

© Copyright, the University Medical Center Utrecht, 2024. All Rights Reserved.

| Task 10: Social cognition - Theory of Mind simple | *Written* |
|---|---|

Number:
Date:

## Who thinks your joke is funny?

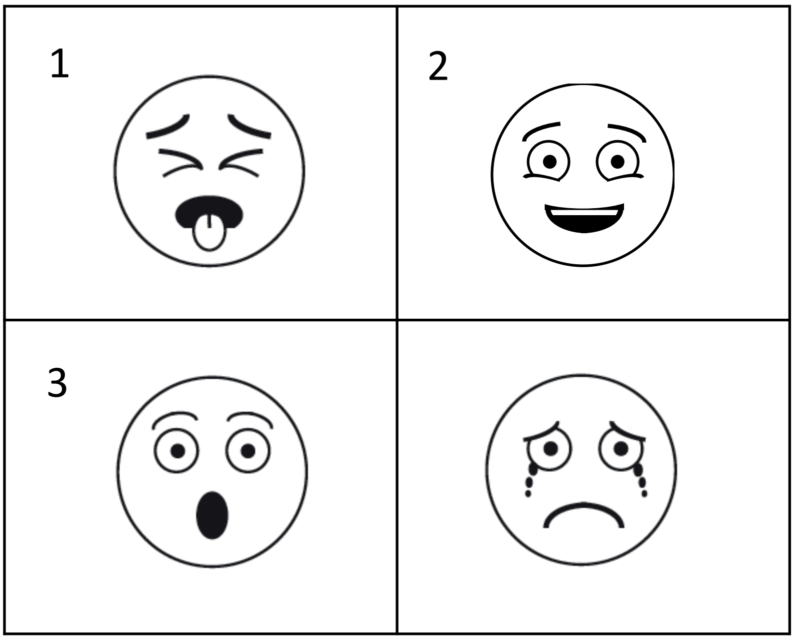


## Who thinks your cake is not delicious?

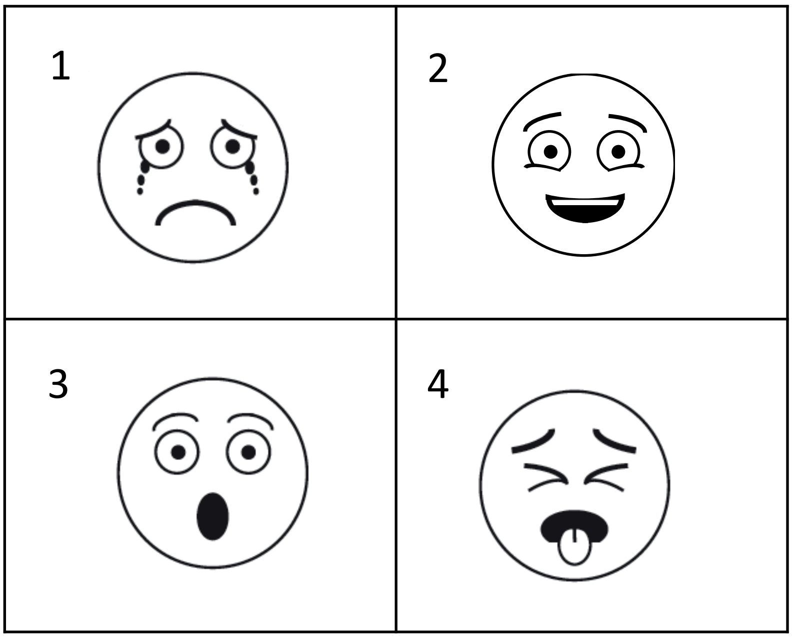

© Copyright, the University Medical Center Utrecht, 2024. All Rights Reserved.

## Task 10: Social cognition - Theory of Mind simple *Written*

Number:
Date:

# Who needs you comfort?

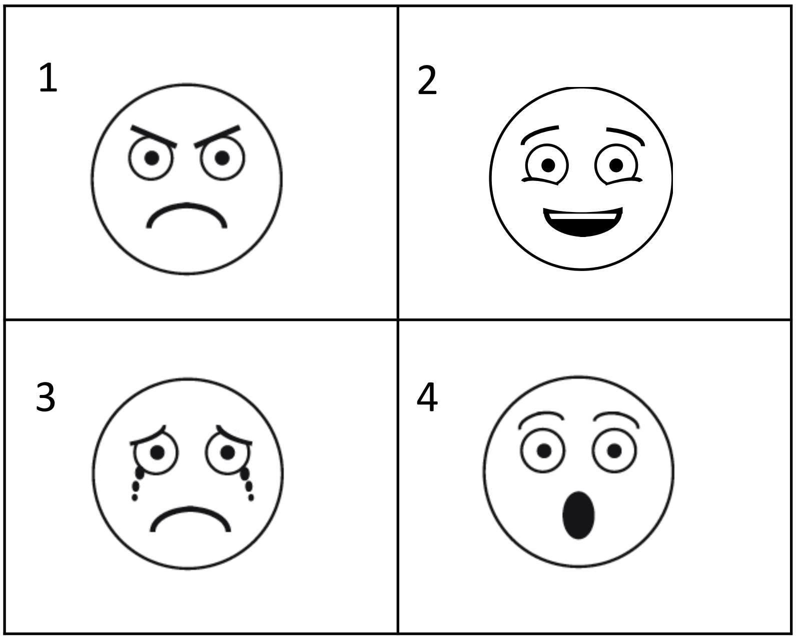


# Who scares you?

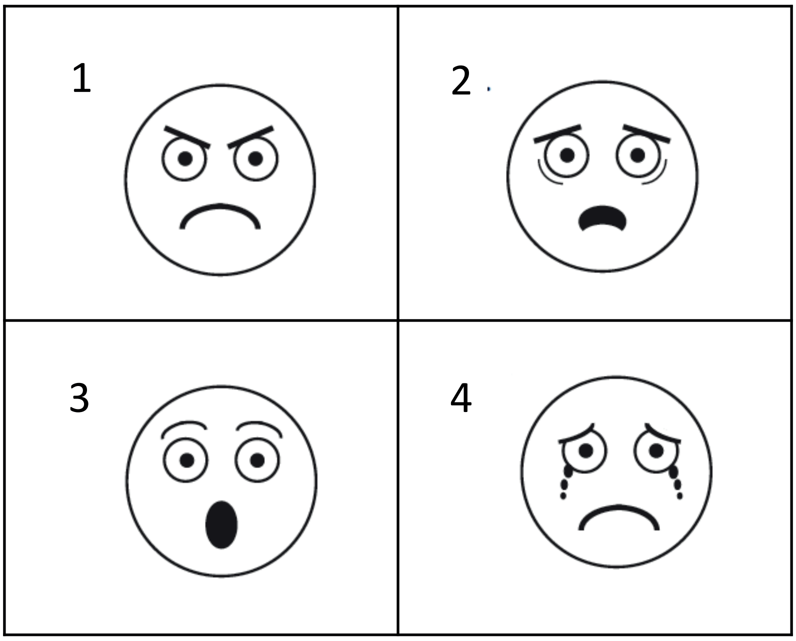

© Copyright, the University Medical Center Utrecht, 2024. All Rights Reserved.

## Task 10: Social cognition - Theory of Mind simple *Written*

Number:
Date:

# Who did you just surprise?

| | |
|---|---|
| 1 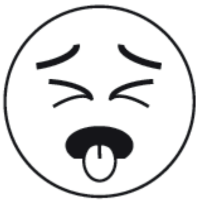 | 2 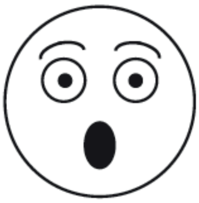 |
| 3 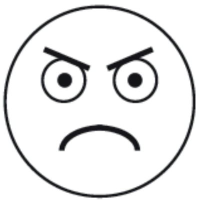 | 4 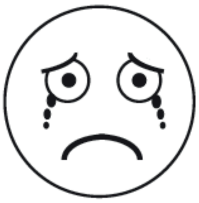 |

# Who did you just show a scary film to?

| | |
|---|---|
| 1 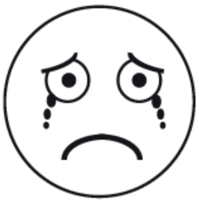 | 2 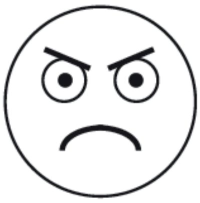 |
| 3 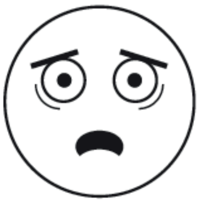 | 4 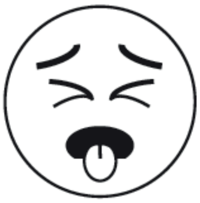 |

© Copyright, the University Medical Center Utrecht, 2024. All Rights Reserved.

## Task 11: Social cognition - Theory of Mind complex *Written*

Number:
Date:

# Example

Answer: ..................................................................................................
..................................................................................................

After hint: ..................................................................................................
..................................................................................................

# Story I

Answer: ..................................................................................................
..................................................................................................

After hint: ..................................................................................................
..................................................................................................

# Story II

Answer: ..................................................................................................
..................................................................................................

After hint: ..................................................................................................
..................................................................................................

# Story III

Answer: ..................................................................................................
..................................................................................................

After hint: ..................................................................................................
..................................................................................................

© Copyright, the University Medical Center Utrecht, 2024. All Rights Reserved.
