# Supplementary material for: Cognitive impairment within and beyond the FTD spectrum in ALS: development of a complementary cognitive screen
Source: J Neurol. 2025 Mar 13;272(4):268. doi: 10.1007/s00415-025-13006-2 (PMC11903523; doi:10.1007/s00415-025-13006-2)
Supplement: Supplementary file 5 — Supplementary file5 (PDF 1592 KB) [file 415_2025_13006_MOESM5_ESM.pdf]

COMPLEMENTARY COGNITIVE ALS SCREEN (C-CAS)
Version A

Date test conducted: ....
Number: ....
Date of birth: .... M/F
Highest completed education: ....
ISCED level: .... (0 - 8)
Left-handed/right-handed: ....

Writing possible: Yes / No
Writing/drawing with dominant hand: Yes / No
Understandable speech: Yes / No
Colour blindness: Yes / No

To calculate a sum score and assess the C-CAS items as 'normal' or 'impaired,' please go to the web application at (https://apps4mnd.com/ccas/).

Comments

....
....
....
....
....
....
....
....
....
....
....
....
....
....
....
....
....
....
....
....
....
....

© Copyright, the University Medical Center Utrecht, 2024. All Rights Reserved.

Task 1: Visuoconstruction - Rey copy *

Do: Place the Rey figure and answer sheet horizontally in front of the participant.
Say: 'You can see a figure here. Your goal is to copy the figure as accurately as possible. It is not a problem if you make a mistake, you can simply cross it out. Take as much time as you need. Do not rotate the figure or your sheet. Are you ready? Then you can start.'

Please note: the task must be completed using a pencil and participants may not rotate the figure or anwser sheet.

Scoring:
The total score is 36 points (maximum 2 per element).
If the participant draws with their non-dominant hand, no deduction is made for sloppiness.
Extra elements drawn are not counted as mistakes.

Scoring	Number of points
Correct element and correct placement	2
Correct element and incorrect placement	1
Element not entirely correct but recognisable and with correct placement	1
Element not entirely correct but recognisable and with incorrect placement	0.5
Element is not present	0

Element	Number of points
1. Vertical cross	
2. Large rectangle	
3. Diagonal cross	
4. Horizontal central line of large rectangle	
5. Vertical central line of large rectangle	
6. Small rectangle	
7. Small horizontal line above small rectangle	
8. Four parallel lines	
9. Small triangle above large rectangle	
10. Small vertical line in large rectangle	
11. Circle with three dots	
12. Five parallel lines	
13. Sides of large triangle to large rectangle	
14. Rhombus	
15. Vertical line within large triangle	
16. Horizontal line within large triangle	
17. Horizontal cross	
18. Square fixed below large rectangle	
Total points for elements	
Any deducted points for sloppiness (max. -2)	
**Total score**	

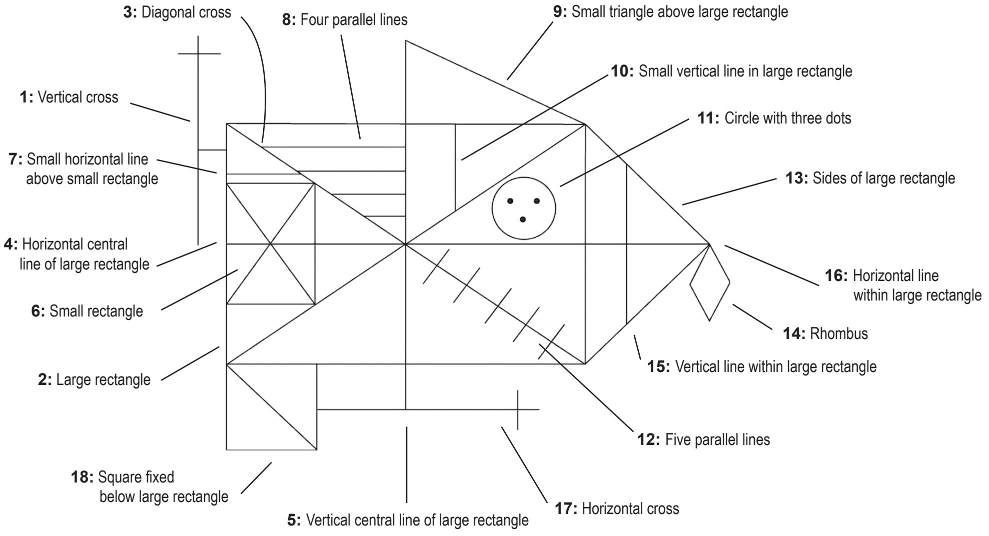

Score 1
(0 - 36)

* *Task cannot be performed if writing is not possible, score = NA*

© Copyright, the University Medical Center Utrecht, 2024. All Rights Reserved.

Task 2: Interference control - Part I **

Say: *'**Name** all the **colors** on this chart as quickly as possible, from left to right. Here's an example.'*
Do: Show the example (blue, green) and check whether the participant has understood the task.
Say: *'We will begin with block **A**, followed by block **B** after a short break.*
*Are you ready? You can begin when I say '**start**'.*

Start the timer after saying the word 'start'.
Write down the time needed to name all colors (seconds, 2 decimals, for block A and B) and record any errors made during the task.
Self-corrections are considered correct.

Please note: the number of errors in this component is not included in the final test results and therefore does not have a score number and does not need to be entered in the web application. However, it is crucial to record the number of errorst, to ensure consistency and accurate interpretation of all tasks.

Color (A) ⬇	Check	Color (A) ⬇	Check	Color (B) ⬇	Check	Color (B) ⬇	Check
yellow		red		red		red	
blue		yellow		blue		yellow	
yellow		blue		blue		green	
blue		green		red		yellow	
green		red		green		blue	
red		red		yellow		red	

Score 2 time block A

Score 2 time block B

Errors (0 - 24)

Task 3: Interference control - Part II **

Say: *'**Read** all the **words** on this chart as quickly as possible, from left to right. Here's an example.'*
Do: Show the example (blue, yellow) and check whether the participant has understood the task.
Say: *'We will begin with block **A**, followed by block **B** after a short break.*
*Are you ready? You can begin when I say '**start**'.*

Start the timer after saying the word 'start'.
Write down the time needed to name all colors (seconds, 2 decimals, for block A and B) and record any errors made during the task.
Self-corrections are considered correct.

Please note: the scores (both times and errors) of this component are not included in the final test results and therefore do not have a score number and do not need to be entered in the web application. However, it is crucial to record these scores, to ensure consistency and accurate interpretation of all tasks.

Color (A) ⬇	Check	Color (A) ⬇	Check	Color (B) ⬇	Check	Color (B) ⬇	Check
yellow		red		red		green	
yellow		green		red		blue	
yellow		green		yellow		blue	
green		yellow		green		yellow	
green		red		green		blue	
green		red		blue		green	

Time block A

Time block B

Errors (0 - 24)

*** Task cannot be performed if a person has incomprehensible articulation (e.g. cannot pronounce colors clearly enough), score = NA*

© Copyright, the University Medical Center Utrecht, 2024. All Rights Reserved.

Task 4: Interference control - Part III **

Say: *‘**Name** all the **colors** on this chart as quickly as possible, so do not read the words! Here’s an example.’*
Do: Show the example (blue, red) and check whether the participant has understood the task.
Say: *‘We will begin with block **A**, followed by block **B** after a short break.*
*Are you ready? You can begin when I say ‘**start**’.*

Start the timer after saying the word ‘start’.
Write down the time needed to name all colors (seconds, 2 decimals, for block A and B) and record any errors made during the task.
Self-corrections are considered correct.

Color (A) ⬇	Check	Color (A) ⬇	Check	Color (B) ⬇	Check	Color (B) ⬇	Check
green		blue		yellow		yellow	
red		blue		red		green	
blue		red		blue		yellow	
yellow		red		green		blue	
green		green		red		red	
green		blue		blue		blue	

Score 3 time block A

Score 3 time block B

Score 4 errors (0 - 24)

Task 5: Interference control - Part IV **

Say: *‘**Name** all the **colors** on this chart as quickly as possible, so do not read the words unless there’s a box around the word. Then read the word instead of the colour. Here’s an example.’*
Do: Show the example (green, yellow) and check whether the participant has understood the task.
Say: *‘We will begin with block **A**, followed by block **B** after a short break.*
*Are you ready? You can begin when I say ‘**start**’.*

Start the timer after saying the word ‘start’.
Write down the time needed to name all colors (seconds, 2 decimals, for block A and B) and record any errors made during the task.
Self-corrections are considered correct.

Please note: the times needed for this component are not included in the final test results and therefore do not have a score number and do not need to be entered in the web application. However, recording these times is important to ensure that participants complete the task as quickly as possible, ensuring consistency and accurate interpretation of all tasks.

Color (A) ⬇	Check	Color (A) ⬇	Check	Color (B) ⬇	Check	Color (B) ⬇	Check
yellow		green		green		yellow	
blue		yellow		blue		yellow	
red		green		green		blue	
red		green		green		green	
blue		yellow		yellow		blue	
blue		yellow		yellow		red	

Time block A

Time block B

Score 5 errors (0 - 24)

*** Task cannot be performed if a person has incomprehensible articulation (e.g. cannot pronounce colors clearly enough), score = NA*

© Copyright, the University Medical Center Utrecht, 2024. All Rights Reserved.

Task 6: Body orientation - Part I

☐ Spoken ☐ Written

Say, if spoken:

*‘Name the color of the **left hand** as quickly as possible if you see an **L**, and the color of the **right hand** if you see an **R**. You will see the line drawing of the figures from behind. Here’s an example.’*

Say if written:

*‘Enter a cross on the **left hand** as quickly as possible if you see an **L**, and on the **right hand** if you see an **R**. You will see the line drawing of the figures from behind. Here’s an example.’*

Do: Show the example (red, red) and check whether the participant has understood the task.

Say: *‘Are you ready? You can begin when I say ‘**start**’.’*

Color (I)	Check
blue	
blue	
red	
blue	
red	
red	
blue	
red	

Time

Score 6 (0-8)

Start the timer after saying the word ‘start’. Write down the time needed in seconds (2 decimals).
Award a point for each correctly named color or entered cross. Self-corrections are considered correct.

Please note: the time needed for this component is not included in the final test results and therefore does not have a score number and does not need to be entered in the web application. However, it is important to record the time to ensure that the participant completes the task as quickly as possible.

Task 7: Body orientation - Part II

☐ Spoken ☐ Written

Say, if spoken:

*‘Again, name the color of the **left hand** as quickly as possible if you see an **L**, and the color of the **right hand** if you see an **R**. Please note, you will now see the line drawing of the figures **from the front**! Here’s an example.’*

Say if written:

*‘Again, enter a cross on the **left hand** as quickly as possible if there’s an **L**, and on the **right hand** if you see an **R**. Please note, you will now see the line drawing of the figures **from the front**! Here’s an example.’*

Do: Show the example (red, blue) and check whether the participant has understood the task.

Say: *‘Are you ready? You can begin when I say ‘**start**’.’*

Color (II)	Check
blue	
blue	
red	
red	
red	
red	
red	
blue	

Time

Score 7 (0-8)

Start the timer after saying the word ‘start’. Write down the time needed in seconds (2 decimals).
Award a point for each correctly named color or entered cross. Self-corrections are considered correct.

Please note: the time needed for this component is not included in the final test results and therefore does not have a score number and does not need to be entered in the web application. However, it is important to record the time to ensure that the participant completes the task as quickly as possible.

© Copyright, the University Medical Center Utrecht, 2024. All Rights Reserved.

Task 8: Body orientation - Part III

☐ Spoken ☐ Written

Say, if spoken:

*'Again, name the color of the **left hand** as quickly as possible if you see an **L**, and the color of the **right hand** if you see an **R**. Please note, you will now see the line drawing of the figures **sometimes** from the back and **sometimes** from the front! Here's an example.'*

Say if written:

*'Again, enter a cross on the **left hand** as quickly as possible if you see an **L**, and on the **right hand** if you see an **R**. Please note, you will now see the line drawing of the figures **sometimes** from the back and sometimes from the front! Here's an example.'*

Do: Show the example (blue, red) and check whether the participant has understood the task.

Say: *'Are you ready? You can begin when I say '**start**'.'*

Color (III)	Check
blue	
red	
blue	
red	
blue	
blue	
red	
blue	

Time

Score 8 (0 - 8)

Start the timer after saying the word 'start'. Write down the time needed in seconds (2 decimals). Award a point for each correctly named color or entered cross. Self-corrections are considered correct.

Please note: the time needed for this component is not included in the final test results and therefore does not have a score number and does not need to be entered in the web application. However, it is important to record the time to ensure that the participant completes the task as quickly as possible.

Task 9: Social cognition - Emotion recognition

☐ Spoken ☐ Written

Say if spoken:

'In the following task, you will see six faces displaying an emotion. Please indicate the emotion that best matches each face. Are you ready? Then we will begin.'

Say if written:

'In the following task, you will see six faces displaying an emotion. Please circle the emotion that best matches each face. Are you ready? Then we will begin.'

Face	Emotion	Check
1	Surprised	
2	Angry	
3	Afraid	
4	Happy	
5	Sad	
6	Disgust	

Award a point for each correct answer. (maximum score = 6)

Score 9 (0 - 6)

Task 10: Social cognition - Theory of Mind simple

☐ Spoken ☐ Written

Say if spoken:

'In the following task you need to indicate which face (1, 2, 3 or 4) corresponds to the action. Are you ready? Then we will begin.'

Say if written:

'In the following task you need to circle the number of the face that corresponds to the action. Are you ready? Then we will begin.'

Action	Face	Check
Who thinks your joke is funny?	2	
Who thinks your cake is not delicious?	4	
Who needs your comfort?	3	
Who scares you?	1	
Who did you just surprise?	2	
Who did you just show a scary film to?	3	

Award a point for each correct answer. (maximum score = 6)

Score 10 (0 - 6)

© Copyright, the University Medical Center Utrecht, 2024. All Rights Reserved.

Task 11: Social cognition - Theory of Mind complex ☐ Spoken ☐ Written

Say: *'This test comprises three stories in which an implicit message is given. You should try to work out what this message is. Here's an example.'*

Example
Say: *Jacob is going to the supermarket with his mother. They arrive at the sweets section and Jacob says: 'Look, those sweets look very good.'*
Question: *What does Jacob really want to say when he says this?*

Answer:
Jacob wants to say *'Please, mum, buy me some sweets'* .

Continue with the hint if the answer is **not** correct (*Jacob continues to say: 'I'm hungry, mum'*), followed by the same question: 'What did Jacob actually mean when he said that?'

Do: Check whether the participant has understood the task.

The aim is for the participant to identify this implicit message. Read each story and write down the answer (if spoken) or have the participant write down their answer (if written).

Scoring:
If the participant cannot provide the correct answer, give an extra hint.
Tick the box of the answer that most closely matches the participant's answer.
If the participant understood the implicit message but phrased it differently, count the answer as correct.
If the answer does not match any of the options, write down the answer verbatim.

	**Score**
Correct answer to the first question	2
Correct answer after giving a hint	1
Incorrect answer	0

Story I

Say: *Daniel wants to conduct a work project but Richard, his boss, has asked someone else to do it. Daniel says to him: 'What a shame. I'm not very busy at the moment.'*
Question: *What does Daniel really want to say when he says this?*

Participant's answer:
- ☐ Daniel wants to say '*Please, Richard, change your mind and give the project to me.'* (or an answer similar to this) = correct answer
- ☐ Daniel isn't that busy right now (too literal)
- ☐ I don't know
- ☐ Other: ....
  ....

Hint: *Daniel continues to say: 'This project is ideal for me.'*
Question: *What does Daniel want Richard to do?*

Participant's answer:
- ☐ Daniel wants Richard to change his mind and give him the project (or an answer similar to this) = correct answer
- ☐ The project aligns with Daniel's work (too literal)
- ☐ I don't know
- ☐ Other: ....
  ....

Score (0 - 2)

© Copyright, the University Medical Center Utrecht, 2024. All Rights Reserved.

Story II

Say: *Paul has to go to an interview and he is running late. While he is cleaning his shoes, he says to his wife, Julia: 'I want to wear this blue shirt, but it's very wrinkled.'*
Question: *What does Paul really want to say when he says this?*

Participant's answer:
- ☐ Paul wants to say '*Could you iron the shirt for me, please?*' (or an answer similar to this) = correct answer
- ☐ Paul thinks his shirt is wrinkled (too literal)
- ☐ I don't know
- ☐ Other: ....
  ....

Hint: *Paul continues to say: It's in the basket of clothes to be ironed'*
Question: *What does Paul want Julia to do?*

Participant's answer:
- ☐ Paul wants Julia to iron his shirt (or an answer similar to this) = correct answer
- ☐ Paul wants Julia to get the basket with clothes for ironing (too literal)
- ☐ I don't know
- ☐ Other: ....
  ....

Score (0 - 2) ☐

Story III

Say: *Lucy has no money, but she wants to go out tonight. Lucy knows that David just got paid. She says to him: 'I don't have any money; things are so expensive nowadays.'*
Question: *What does Lucy really want to say when she says this?*

Participant's answer:
- ☐ Lucy wants to say '*Can you loan me some money, David?*' or '*Would you like to take me out tonight?*' (or an answer similar to this) = correct answer
- ☐ Lucy thinks everything is so expensive nowadays (too literal)
- ☐ I don't know
- ☐ Other: ....
  ....

Hint: *Lucy continues to say: 'Well, I guess I'll have to stay in tonight.'*
Question: *What does Lucy want David to do?*

Participant's answer:
- ☐ Lucy wants David to loan her money, or to take her out (or an answer similar to this) = correct answer
- ☐ Comfort her (too literal)
- ☐ I don't know
- ☐ Other: ....
  ....

Score (0 - 2) ☐

Theory of Mind complex score

Score story I ☐ + Score story II ☐ + Score story III ☐ = **Score 11 (0 - 6)** ☐

© Copyright, the University Medical Center Utrecht, 2024. All Rights Reserved.

Task 12: Conflicting instructions

Say: *'For the next task I would like to ask you to place your hand on the table. I will do the same. Tap twice when I tap once.'*
(Do a series of three exercises: 1-1-1)
Say: *'And tap once when I tap twice.'*
(Do a series of three exercises: 2-2-2)

Say: *Say: 'Are you ready? Then we will begin.'*

Do: Now tap the following series: 1-1-2-1-2-2-2-1-1-2.

Ensure any rings on the hand used to tap are removed by both the tester and participant. If the participant cannot tap with one hand, tapping should be done using a foot by both the participant and the tester. Sit opposite each other during the test so the participant can clearly see the tester's foot. If it is not possible for the participant to tap their foot, then the task is not possible, and the score should be marked as "NA."

	**Score**
faultless (10 taps correct)	3
1-2 mistakes	2
> 2 mistakes	1
followed the example rhythm at least four times	0

Score 12 (0 - 3)

13: Action restraint

Say: *'Again tap twice when I tap once.'*
(Do a series of three exercises: 1-1-1)
Say: *'And **do not** tap when I tap twice.'*
(Do a series of three exercises: 2-2-2)

Say: *Say: 'Are you ready? Then we will begin.'*

Do: Now tap the following series: 1-1-2-1-2-2-2-1-1-2.

Ensure any rings on the hand used to tap are removed by both the tester and participant. If the participant cannot tap with one hand, tapping should be done using a foot by both the participant and the tester. Sit opposite each other during the test so the participant can clearly see the tester's foot. If it is not possible for the participant to tap their foot, then the task is not possible, and the score should be marked as "NA."

	**Score**
faultless	3
1-2 mistakes	2
> 2 mistakes	1
followed the example rhythm at least four times	0

Score 13 (0 - 3)

© Copyright, the University Medical Center Utrecht, 2024. All Rights Reserved.

Task 14: Visuoconstruction - Rey recall *

Do: Place the answer sheet horizontally in front of the participant.
Say: *'You had to copy a figure at the beginning of this test. Can you try to draw that figure again from memory. If you remember a particular detail but do not recall exactly where it was, you should still place it somewhere.'*

Please note: the task must be completed using a pencil and participants may not rotate the anwser sheet.

Scoring:
The total score is 36 points (maximum 2 per element).
If the participant draws with their non-dominant hand, no deduction is made for sloppiness.
Extra elements drawn are not counted as mistakes.

Scoring	Number of points
Correct element and correct placement	2
Correct element and incorrect placement	1
Element not entirely correct but recognisable and with correct placement	1
Element not entirely correct but recognisable and with incorrect placement	0.5
Element is not present	0

Element	Number of points
1. Vertical cross	
2. Large rectangle	
3. Diagonal cross	
4. Horizontal central line of large rectangle	
5. Vertical central line of large rectangle	
6. Small rectangle	
7. Small horizontal line above small rectangle	
8. Four parallel lines	
9. Small triangle above large rectangle	
10. Small vertical line in large rectangle	
11. Circle with three dots	
12. Five parallel lines	
13. Sides of large triangle to large rectangle	
14. Rhombus	
15. Vertical line within large triangle	
16. Horizontal line within large triangle	
17. Horizontal cross	
18. Square fixed below large rectangle	
Total points for elements	
Any deducted points for sloppiness (max. -2)	
**Total score**	

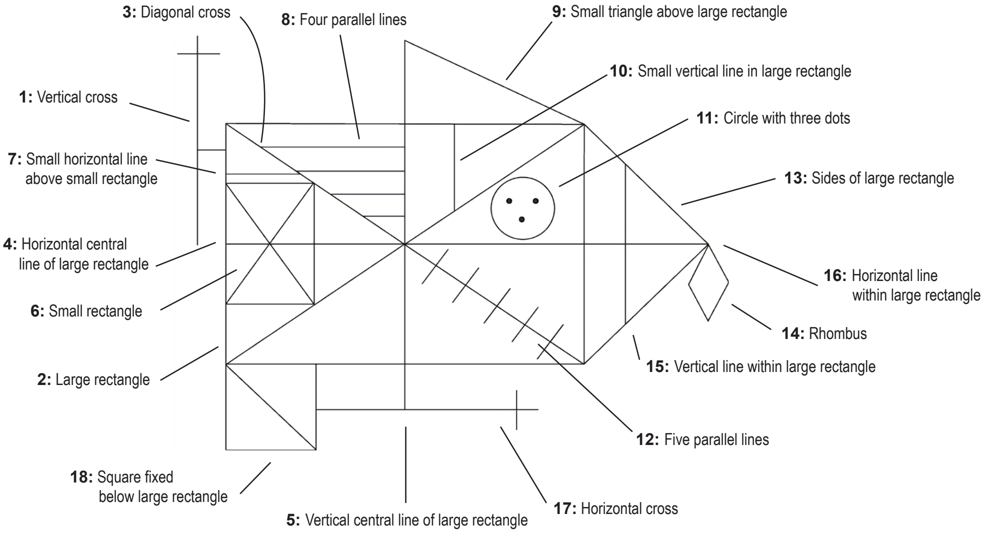

Score 14 (0 - 36)

* Task cannot be performed if writing is not possible, score = NA

© Copyright, the University Medical Center Utrecht, 2024. All Rights Reserved.

© Copyright, the University Medical Center Utrecht, 2024. All Rights Reserved.

14: Visuoconstruction - Recall *

Number:
Date:

** Task cannot be performed if writing is not possible, score = NA*

© Copyright, the University Medical Center Utrecht, 2024. All Rights Reserved.

1: Visuoconstruction - Copy *

Number:
Date:

** Task cannot be performed if writing is not possible, score = NA*
