# Supplementary material for: Cognitive impairment within and beyond the FTD spectrum in ALS: development of a complementary cognitive screen
Source: J Neurol. 2025 Mar 13;272(4):268. doi: 10.1007/s00415-025-13006-2 (PMC11903523; doi:10.1007/s00415-025-13006-2)
Supplement: Supplementary file 6 — Supplementary file6 (PDF 2296 KB) [file 415_2025_13006_MOESM6_ESM.pdf]

COMPLEMENTARY COGNITIVE ALS SCREEN (C-CAS)
Manual – Version A

Required equipment:

- C-CAS form – A
- C-CAS appendix – A
- Clipboard
- Stopwatch
- Pens and pencils
- C-CAS appendix for written answers – A (if someone is unable to speak, use this document for task 6 to 11)

BASIC DETAILS

Collect the following basic details to determine whether the C-CAS items are 'normal' or 'impaired': date of birth, date the test was conducted, participant's gender, and educational level according to the International Standard Classification of Education (ISCED 2011).

The C-CAS can be conducted in full if the participant is color blind, provided they can distinguish the colors. If the participant is unable to distinguish colors, conduct the tasks Body orientation part I, II, and III in writing instead of spoken. The tasks Interference control part I, II, III, and IV cannot be conducted if the participant is unable to distinguish colors.

TASK 1: VISUOCONSTRUCTION - REY COPY

If someone is unable to draw (with either hand) due to hand motor problems, this task cannot be carried out. Please ensure that you have the Rey figure printed for this task.

Test: Place the figure and answer sheet (task 1 found at the end of the C-CAS) horizontally in front of the participant. Say: *'You can see a figure here. Your goal is to copy the figure as accurately as possible. It is not a problem if you make a mistake, you can simply cross it out. Take as much time as you need. Do not rotate the figure or your sheet. Are you ready? Then you can start.'*

Ensure the participants use a pencil for this task. It is not allowed to use an eraser; if anyone wants to use an eraser, instruct them to cross out any mistakes. Participants may not rotate the form. If a participant attempts to rotate the form, remind them of the rules mentioned above.

Scoring: Total score is 36 points, with a maximum of 2 points per element. If the participant draws with their non-dominant hand, no deduction is made for sloppiness. Extra elements drawn are not counted as mistakes. Each of the 18 elements (table 2) is scored according to table 1.

© Copyright, the University Medical Center Utrecht, 2024. All Rights Reserved.

Table 1

**Scoring**	**Number of points**
Correct element and correct placement	2
Correct element, incorrect placement	1
Element not entirely correct but recognisable and with correct placement	1
Element not entirely correct but recognisable and with incorrect placement	0.5
Element is not present	0

Table 2

**Element**	**Number of points**
1. Vertical cross	
2. Large rectangle	
3. Diagonal cross	
4. Horizontal central line of large rectangle	
5. Vertical central line of large rectangle	
6. Small rectangle	
7. Small horizontal line above small rectangle	
8. Four parallel lines	
9. Small triangle above large rectangle	
10. Small vertical line in large rectangle	
11. Circle with three dots	
12. Five parallel lines	
13. Sides of large triangle to large rectangle	
14. Rhombus	
15. Vertical line within large triangle	
16. Horizontal line within large triangle	
17. Horizontal cross	
18. Square fixed below large rectangle	
Total points for elements	
Any deducted points for sloppiness (max. -2)	
**Total score**	

© Copyright, the University Medical Center Utrecht, 2024. All Rights Reserved.

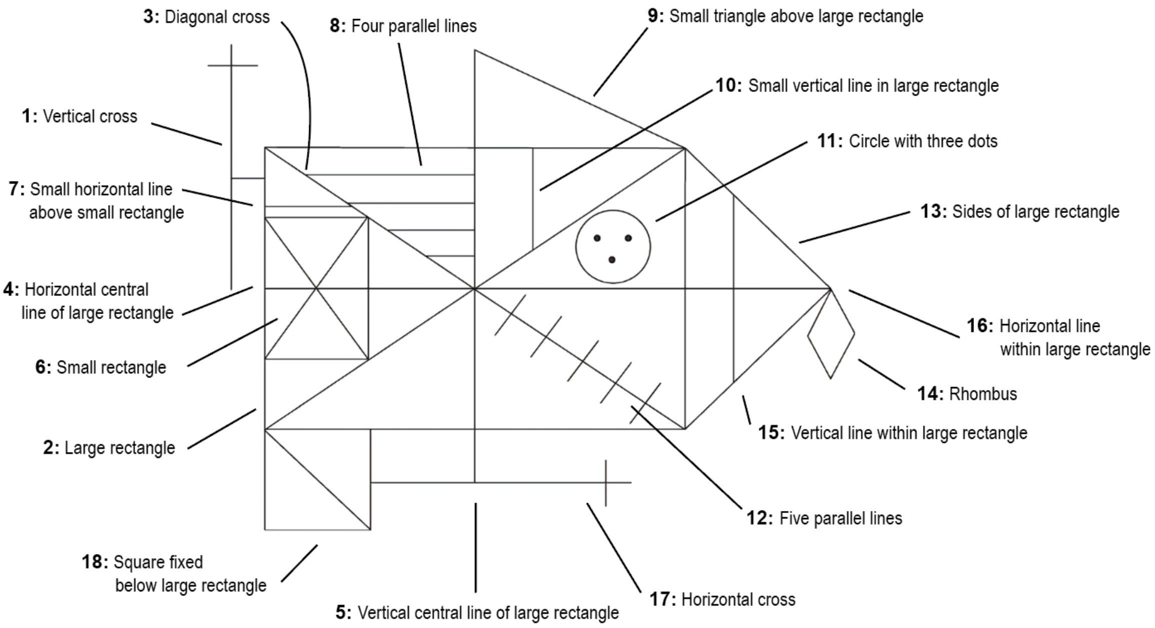

Additional explanation for the scoring: Extra elements drawn, such as 5 parallel lines instead of 4 parallel lines, are not counted as incorrect (no points deducted). However, if someone repeatedly makes these types of mistakes, you may decide to deduct a point. A maximum deduction of -2 can be subtracted from the total score for overall sloppiness. This deduction does not apply if a person has impaired hand function due to the disease and therefore cannot draw accurately.
All elements are graphically displayed for better understanding at the end of this manual (appendix 1).

TASK 2: INTERFERENCE CONTROL - PART I

If a person is unable to speak, the tasks Interference control part I, II, III and IV cannot be conducted. You can leave the corresponding field in the web application empty (for more information about the web application see the last page of this document).

Test: Place the chart with the example (task 2) in front of the participant. Say: *'Name all the colors on this chart as quickly as possible, from left to right. Here's an example.'* Show the example (blue, green) and check whether the participant has understood the task. Say: *'We will begin with block A, followed by block B after a short break. Are you ready? You can begin when I say 'start'.'* Then rotate the chart to block A, say '*start*' straight away and start the stopwatch. Prevent the participant from looking at the chart before you give the starting signal. This also applies to block B.

Scoring: Write down the time needed to name all colors (seconds, 2 decimals, for block A and B) and record any errors made during the task. Self-corrections are considered correct. Self-corrections made outside the time, so after you stopped the stopwatch, will be assessed as errors.

Please note: the number of errors in this component is not included in the final test results and therefore does not have a score number and does not need to be entered in the web application. However, it is crucial to record the number of errors, to ensure consistency and accurate interpretation of all tasks.

© Copyright, the University Medical Center Utrecht, 2024. All Rights Reserved.

TASK 3: INTERFERENCE CONTROL - PART II

If a person is unable to speak due to bulbar motor problems, the tasks Interference control part I, II, III and IV cannot be conducted.

Test: Place the chart with the example (task 3) in front of the participant. Say: *'Read all the words on this chart as quickly as possible, from left to right. Here's an example.'* Show the example (blue, yellow) and check whether the participant has understood the task. Say: *'We will begin with block A, followed by block B after a short break. Are you ready? You can begin when I say 'start'.'* Then rotate the chart to block A, say '*start*' straight away and start the stopwatch. Prevent the participant from looking at the chart before you give the starting signal. This also applies to block B.

Scoring: Write down the time needed to name all colors (seconds, 2 decimals, for block A and B) and record any errors made during the task. Self-corrections are considered correct. Self-corrections made outside the time, so after you stopped the stopwatch, will be assessed as errors.

Please note: the scores (both times and errors) of this component are not included in the final test results and therefore do not have score numbers and do not need to be entered in the web application. However, it is crucial to record these scores, to ensure consistency and accurate interpretation of all tasks.

TASK 4: INTERFERENCE CONTROL - PART III

If a person is unable to speak due to bulbar motor problems, the tasks Interference control part I, II, III and IV cannot be conducted.

Test: Place the chart with the example (task 4) in front of the participant. Say: *'Name all the colors on this chart as quickly as possible, so do not read the words! Here's an example.'* Show the example (blue, red) and check whether the participant has understood the task. Say: *'We will begin with block A, followed by block B after a short break. Are you ready? You can begin when I say 'start'.'* Then rotate the chart to block A, say '*start*' straight away and start the stopwatch. Prevent the participant from looking at the chart before you give the starting signal. This also applies to block B.

Scoring: Write down the time needed to name all colors (seconds, 2 decimals, for block A and B) and record any errors made during the task. Self-corrections are considered correct. Self-corrections made outside the time, so after you stopped the stopwatch, will be assessed as errors.

TASK 5: INTERFERENCE CONTROL - PART IV

If a person is unable to speak due to bulbar motor problems, the tasks Interference control part I, II, III and IV cannot be conducted.

Test: Place the chart with the example (task 5) in front of the participant. Say: *'Name all the colors on this chart as quickly as possible, so don't read the words unless there is a box around the word. Then*

© Copyright, the University Medical Center Utrecht, 2024. All Rights Reserved.

read the word instead of the color. Here's an example.' Show the example (green, yellow) and check whether the participant has understood the task. Say: *'We will begin with block A, followed by block B after a short break. Are you ready? You can begin when I say 'start'.'* Then rotate the chart to block A, say '*start*' straight away and start the stopwatch. Prevent the participant from looking at the chart before you give the starting signal. This also applies to block B.

<u>Scoring:</u> Write down the time needed to name all colors (seconds, 2 decimals, for block A and B) and record any errors made during the task. Self-corrections are considered correct. Self-corrections made outside the time, so after you stopped the stopwatch, will be assessed as errors.

Please note: the times needed for this component are not included in the final test results and therefore do not have score numbers and do not need to be entered in the web application. However, recording these times is important to ensure that participants complete the task as quickly as possible, ensuring consistency and accurate interpretation of all tasks.

TASK 6: BODY ORIENTATION - PART I

<u>Test</u>: Place the chart with the example (task 6) in front of the participant. Say:

> **If spoken**: *'Name the color of the left hand as quickly as possible if you see an L, and the color of the right hand if you see an R. You will see the line drawings of the figures from behind. Here's an example.'*
> **If written:** *'Enter a cross on the left hand as quickly as possible if you see an L, and on the right hand if you see an R. You will see the line drawings of the figures from behind. Here's an example.'.*

Show the example (<u>spoken:</u> red, red) and check whether the participant has understood the task. The example for the written administration is included at the end of the C-CAS appendix. Say: *'Are you ready? You can begin when I say 'start'.'* Then rotate the chart, say '*start*' straight away and start the stopwatch. Prevent the participant from looking at the chart before you give the starting signal.

<u>Scoring:</u> Write down the time needed in seconds (2 decimals). Award a point for each correct element. Consider self-corrections as correct. However, self-corrections made after stopping the stopwatch will be assessed as errors. The correct answers are added at the end of this manual (appendix 2).

Please note: the time needed for this component is not included in the final test results and therefore does not have a score number and does not need to be entered in the web application. However, it is important to record the time to ensure that the participant completes the task as quickly as possible. Pausing the test and restarting it is not allowed.

TASK 7: BODY ORIENTATION - PART II

<u>Test:</u> Place the chart with the example (task 7) in front of the participant. Say:

> **If spoken**: *'Again, name the color of the left hand as quickly as possible if you see an L, and the color of the right hand if you see an R. Please note, you will now see the line drawings of the figures from the front! Here's an example.'*

© Copyright, the University Medical Center Utrecht, 2024. All Rights Reserved.

If written: *'Again, enter a cross on the left hand as quickly as possible if you see an L, and on the right hand if you see an R. Please note, you will now see the line drawings of the figures from the front! Here's an example.'.*

Show the example (spoken: red, blue) and check whether the participant has understood the task. The example for the written administration is included at the end of the C-CAS appendix. Say: *'Are you ready? You can begin when I say 'start'.'* Then rotate the chart, say '*start*' straight away and start the stopwatch. Prevent the participant from looking at the chart before you give the starting signal.

Scoring: Write down the time needed in seconds (2 decimals). Award a point for each correct element. Consider self-corrections as correct. However, self-corrections made after stopping the stopwatch will be assessed as errors. The correct answers are added at the end of this manual (appendix 2).

Please note: the time needed for this component is not included in the final test results and therefore does not have a score number and does not need to be entered in the web application. However, it is important to record the time to ensure that the participant completes the task as quickly as possible. Pausing the test and restarting it is not allowed.

TASK 8: BODY ORIENTATION - PART III

Test: Place the chart with the example (task 8) in front of the participant. Say:

If spoken: *'Again, name the color of the left hand as quickly as possible if you see an L, and the color of the right hand if you see an R. Please note, you will now see the line drawings of the figures sometimes from the back and sometimes from the front! Here's an example.'*

If written: *'Again, enter a cross on the left hand as quickly as possible if you see an L, and on the right hand if you see an R. Please note, you will now see the line drawings of the figures sometimes from the back and sometimes from the front! Here's an example.'.*

Show the example (spoken: blue, red) and check whether the participant has understood the task. The example for the written administration is included at the end of the C-CAS appendix. Say: *'Are you ready? You can begin when I say 'start'.'* Then rotate the chart, say '*start*' straight away and start the stopwatch. Prevent the participant from looking at the chart before you give the starting signal.

Scoring: Write down the time needed in seconds (2 decimals). Award a point for each correct element. Consider self-corrections as correct. However, self-corrections made after stopping the stopwatch will be assessed as errors. The correct answers are added at the end of this manual (appendix 2).

Please note: the time needed for this component is not included in the final test results and therefore does not have a score number and does not need to be entered in the web application. However, it is important to record the time to ensure that the participant completes the task as quickly as possible. Pausing the test and restarting it is not allowed.

TASK 9: SOCIAL COGNITION - EMOTION RECOGNITION

Test: Place the right chart (task 9) in front of the participant. Say:

If spoken: *'In the following task, you will see six faces displaying an emotion. Please indicate the emotion that best matches each face. Are you ready? Then we will begin.'*

© Copyright, the University Medical Center Utrecht, 2024. All Rights Reserved.

If written: *'In the following task, you will see six faces displaying an emotion. Please circle the emotion that best matches each face. Are you ready? Then we will begin.'*

Scoring: Award a point for each good answer (maximum score = 6).

TASK 10: SOCIAL COGNITION - THEORY OF MIND SIMPLE

Test: Place the right chart (task 10) in front of the participant. Say:

If spoken: *'In the following task you need to indicate which face (1, 2, 3 or 4) corresponds to the action. Are you ready? Then we will begin.'*
If written: *'In the following task you need to circle the number of the face that corresponds to the action. Are you ready? Then we will begin.'*

Scoring: Award a point for each good answer (maximum score = 6).

TASK 11: SOCIAL COGNITION - THEORY OF MIND COMPLEX

Test: Say: *'This test comprises three stories in which an implicit message is given. You should try to work out what this message is* (you can say it or write it down). *Here's an example.'*

Example:
Say: 'Jacob is going to the supermarket with his mother. They arrive at the sweets section and Jacob says: 'Look, those sweets look very good.'
Question: 'What does Jacob really want to say when he says this?'.
Answer: Jacob wants to say 'Please, mum, buy me some sweets'.
Continue with the hint if the answer is not correct (**Hint**: Jacob continues to say: 'I'm hungry, mum'), followed by the same **question**: 'What did Jacob actually mean when he said that?'.

Check whether the participant has understood the task.

This test comprises three stories, each with an implicit message. The aim is for the participant to identify this implicit message. Read each story and write down the answer (if spoken) or have the participant write down their answer (if written). If you are not immediately sure whether the answer is correct, provide a hint to confirm and check afterwards whether the initial answer was correct. In such cases, still award 2 points if the answer was correct. If someone writes the answers, check for legibility and consider giving a hint after the first answer if needed.

Scoring: If the participant cannot provide the correct answer, give the hint. Tick the box of the answer that most closely matches the participant's answer. If the participant understood the implicit message but phrased it differently, count the answer as correct. If the answer does not match any of the options, write down the answer verbatim.

	**Score**
Correct answer to the first question	2
Correct answer after giving a hint	1
Incorrect answer	0

© Copyright, the University Medical Center Utrecht, 2024. All Rights Reserved.

Additional examples scoring	
Story 1	
Correct answer: 2 points (or 1 point if this answer is only given after the hint)	***Incorrect answer: 0 points***
- 'That he really wanted that job'<br>- 'That he currently does have time to lead the project'<br>- 'I have time to do that project. Have you thought of me?'<br>- 'Give me that job'	- 'He is not busy'
Story 2	
- 'That the partner/Julia needs to iron better'	- 'Julia has to help to find some other clothes'<br>- 'The clothes are wrinkled'
Story 3	
- 'Still go out and the partner/David has to pay this time'<br>- 'To borrow me some money'	- 'I actually don't have enough money'<br>- 'Really wanted to be invited'<br>- 'That David comes along'

TASK 12: CONFLICTING INSTRUCTIONS

Test:
Say: *'For the next task I would like to ask you to place your hand on the table. I will do the same. Tap twice when I tap once.'*
(Do a series of three exercises: 1-1-1)
Say: *'And tap once when I tap twice.'*
(Do a series of three exercises: 2-2-2)
Say: *'Are you ready? Then we will begin.'*
Do: Now tap the following series: 1-1-2-1-2-2-2-1-1-2.

Ensure any rings on the hand used to tap are removed by both the tester and participant to avoid distraction. If the participant cannot tap with one hand, tapping should be done using a foot by both the participant and the tester. Sit opposite each other during the test so the participant can clearly see the tester's foot. If it is not possible for the participant to tap their foot, then the task is not possible, and the score should be marked as "NA."

Scoring: Keep track of the mistakes made by the participant during the test. This is important to determine whether the participant will receive 1 or 0 points. If the participant consistently imitates the test leader (copies the example rhythm and does this at least four times), they will receive 0 points. If the participant imitates the test leader less than four times or makes another kind of mistake, they will receive 1 point. If the participant raises their hand to tap but then decides not to tap, this should not be assessed as a mistake.

	**Score**
Faultless (10 taps correct)	3
1 - 2 mistakes	2
>2 mistakes	1
Followed the example rhythm at least four times	0

© Copyright, the University Medical Center Utrecht, 2024. All Rights Reserved.

TASK 13: ACTION RESTRAINT

Test:
Say: *'Again tap twice when I tap once.'*
(Do a series of three exercises: 1-1-1)
Say: *'And don't tap when I tap twice.'*
(Do a series of three exercises: 2-2-2)
Say: *'Are you ready? Then we will begin.'*
Do: Now tap the following series: 1-1-2-1-2-2-2-1-1-2.

Ensure any rings on the hand used to tap are removed by both the tester and participant to avoid distraction. If the participant cannot tap with one hand, tapping should be done using a foot by both the participant and the tester. Sit opposite each other during the test so the participant can clearly see the tester's foot. If it is not possible for the participant to tap their foot, then the task is not possible, and the score should be marked as "NA."

Scoring: Keep track of the mistakes made by the participant during the test. This is important to determine whether the participant will receive 1 or 0 points. If the participant consistently imitates the test leader (copies the example rhythm and does this at least four times), they will receive 0 points. If the participant imitates the test leader less than four times or makes another kind of mistake, they will receive 1 point. Participants using the instructions from task 12 in task 13 will result in 5 mistakes, leading to a score of 1 point. If the participant raises their hand to tap but then decides not to tap, this should not be assessed as a mistake.

	**Score**
Faultless (10 taps correct)	3
1 - 2 mistakes	2
>2 mistakes	1
Followed the example rhythm at least four times	0

TASK 14: VISUOCONSTRUCTION - REY RECALL

If task 1 (Visuoconstruction - Rey copy) has not been conducted, this task will not be conducted either.

Test: Place the answer sheet (task 14 found at the end of the C-CAS) horizontally in front of the participant. Say: *'You had to copy a figure at the beginning of this test. Can you try to draw that figure again from memory. If you remember a particular detail but do not recall exactly where it was, you should still place it somewhere.'*

Ensure the participants use a pencil for this task. It is not allowed to use an eraser; if anyone wants to use an eraser, instruct them to cross out any mistakes. Participants may not rotate the form. If a participant attempts to rotate the form, remind them that that is not allowed.
Scoring: Total score is 36 points, with a maximum of 2 points per element. If the participant draws with their non-dominant hand, no deduction is made for sloppiness. Extra elements drawn are not counted as mistakes. Each of the 18 elements (table 2) is scored according to table 1.

© Copyright, the University Medical Center Utrecht, 2024. All Rights Reserved.

Table 1

**Scoring**	**Number of points**
Correct element and correct placement	2
Correct element, incorrect placement	1
Element not entirely correct but recognisable and with correct placement	1
Element not entirely correct but recognisable and with incorrect placement	0.5
Element is not present	0

Table 2

**Element**	**Number of points**
1. Vertical cross	
2. Large rectangle	
3. Diagonal cross	
4. Horizontal central line of large rectangle	
5. Vertical central line of large rectangle	
6. Small rectangle	
7. Small horizontal line above small rectangle	
8. Four parallel lines	
9. Small triangle above large rectangle	
10. Small vertical line in large rectangle	
11. Circle with three dots	
12. Five parallel lines	
13. Sides of large triangle to large rectangle	
14. Rhombus	
15. Vertical line within large triangle	
16. Horizontal line within large triangle	
17. Horizontal cross	
18. Square fixed below large rectangle	
Total points for elements	
Any deducted points for sloppiness (max. -2)	
**Total score**	

Additional explanation for the scoring: Extra elements drawn, such as 5 parallel lines instead of 4 parallel lines, are not counted as incorrect (no points deducted). However, if someone repeatedly makes these types of mistakes, you may decide to deduct a point. A maximum deduction of -2 can be subtracted from the total score for overall sloppiness. This deduction does not apply if a person has impaired hand function due to the disease and therefore cannot draw accurately.
All elements are graphically displayed for better understanding at the end of this manual (appendix 1).

© Copyright, the University Medical Center Utrecht, 2024. All Rights Reserved.

C-CAS WEB APPLICATION

A web application has been developed for calculating a sum score and assessing the C-CAS items as ‘normal’ or ‘impaired’. To use the application, enter the C-CAS scores and some basic details (date of birth, test date, gender, and educational level). Based on the normative data from controls, the application generates results. The web application is available at (https://apps4mnd.com/ccas/).

Reference: Michielsen, A., van den Berg, L.H., Westeneng, H.J. (2025). Cognitive impairment within and beyond the FTD spectrum in ALS: development of a complementary cognitive screen. Journal of Neurology.

© Copyright, the University Medical Center Utrecht, 2024. All Rights Reserved.

APPENDIX 1: REY ELEMENTS GRAPHICALLY DISPLAYED

1. Vertical cross 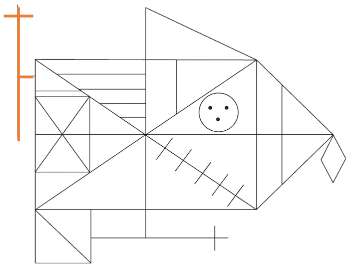	6. Small rectangle 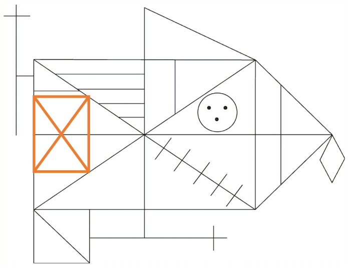
2. Large rectangle 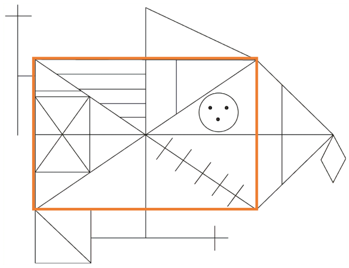	7. Small horizontal line above small rectangle 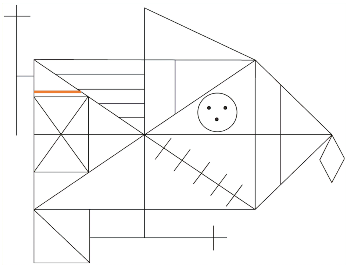
3. Diagonal cross 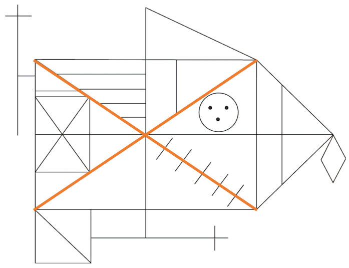	8. Four parallel lines 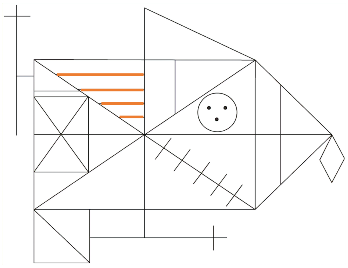
4. Horizontal central line of large rectangle 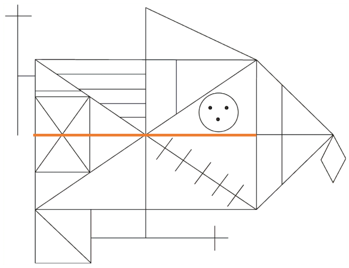	9. Small triangle above large rectangle 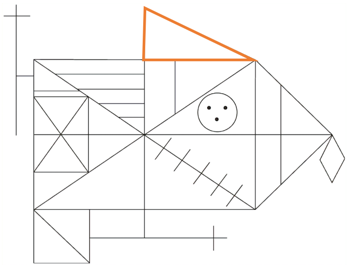
5. Vertical central line of large rectangle 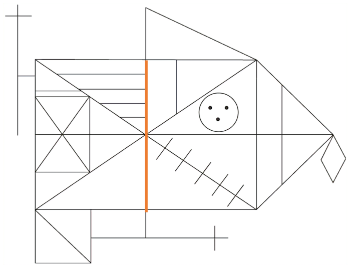	10. Small vertical line in large rectangle 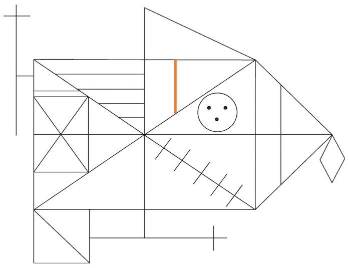

© Copyright, the University Medical Center Utrecht, 2024. All Rights Reserved.

11. Circle with three dots 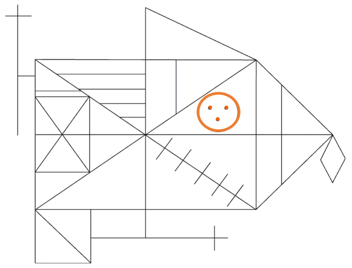	16. Horizontal line within large triangle 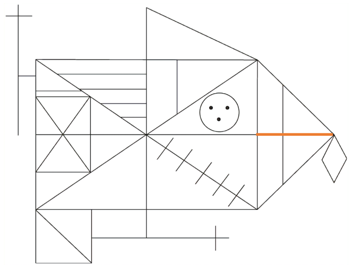
12. Five parallel lines 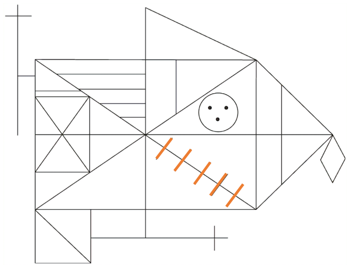	17. Horizontal cross 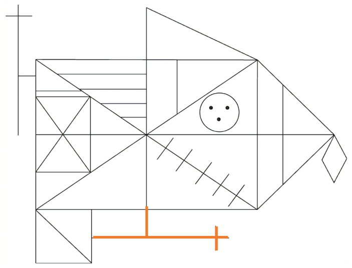
13. Sides of large triangle to large rectangle 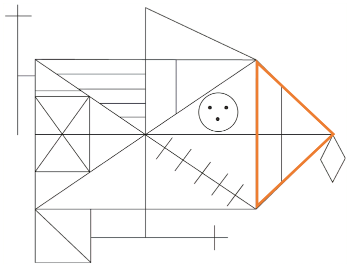	18. Square fixed below large rectangle 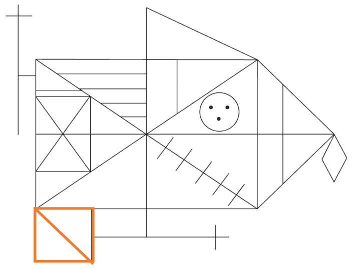
14. Rhombus 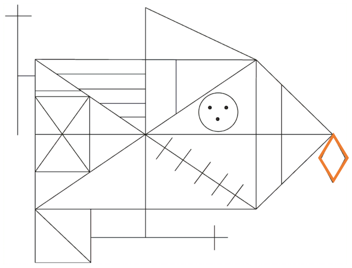	
15. Vertical line within large triangle 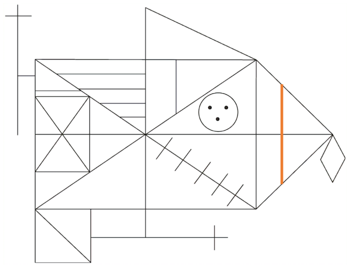	

© Copyright, the University Medical Center Utrecht, 2024. All Rights Reserved.

APPENDIX 2: WRITTEN ANSWERS BODY ORIENTATION TASK

Task 6: Body orientation – Part I

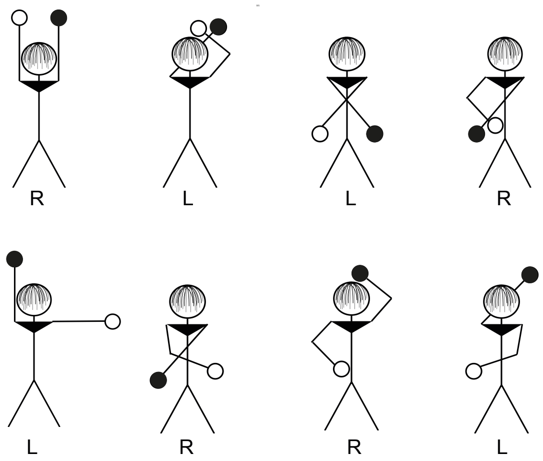

Task 7: Body orientation – Part II

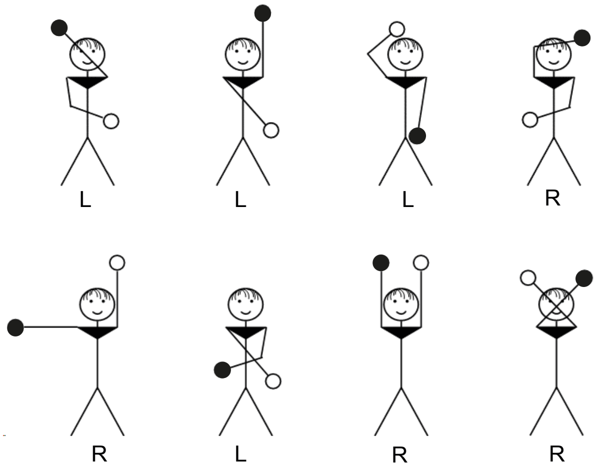

Task 8: Body orientation – Part III

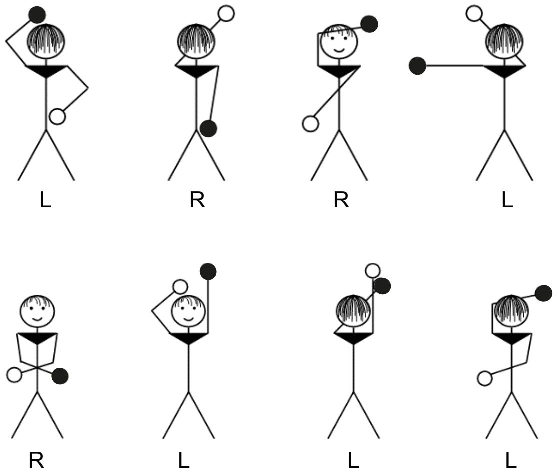

© Copyright, the University Medical Center Utrecht, 2024. All Rights Reserved.
